# Association of glucose-lowering drug target and risk of gastrointestinal cancer: a mendelian randomization study

**DOI:** 10.1186/s13578-024-01214-8

**Published:** 2024-03-19

**Authors:** Yi Yang, Bo Chen, Chongming Zheng, Hao Zeng, Junxi Zhou, Yaqing Chen, Qing Su, Jingxian Wang, Juejin Wang, Yurong Wang, Hongli Wang, Ruxue Jin, Zhiyuan Bo, Gang Chen, Yi Wang

**Affiliations:** 1 Department of Epidemiology and Biostatistics, School of Public Health and Management, Wenzhou Medical University, Wenzhou, 325035 China; 2 Department of Hepatobiliary Surgery, The First Affiliated Hospital of Wenzhou Medical University, Wenzhou, 325035 China; 3 Key Laboratory of Diagnosis and Treatment of Severe Hepato-Pancreatic Diseases of Zhejiang Province, The First Affiliated Hospital of Wenzhou Medical University, Wenzhou, China; 4 Zhejiang-Germany Interdisciplinary Joint Laboratory of Hepatobiliary-Pancreatic Tumor and Bioengineering, Zhejiang, China; 5 Wenzhou Medical University, Wenzhou, China

**Keywords:** Glucose-lowering drug target, Gastrointestinal cancer risk, Causality, Mendelian randomization

## Abstract

**Background & Aims:**

Glucose-lowering drug is associated with various cancers, but the causality with gastrointestinal cancer risk is rarely reported. We aimed to explore the causality between them in this Mendelian randomization (MR) study.

**Methods:**

Two-sample MR, summary-data-based (SMR), mediation MR, and colocalization analyses was employed. Ten glucose-lowering drug targets (PPARG, DPP4, GLP1R, INSR, SLC5A2, ABCC8, KCNJ11, ETFDH, GPD2, PRKAB1) and seven types of gastrointestinal cancer (anal carcinoma, cardia cancer, gastric cancer, hepatocellular carcinoma (HCC), intrahepatic cholangiocarcinoma (ICC), pancreatic cancer, rectum cancer) were included. Patients with gastrointestinal cancers from six different large GWAS databases, including the UK Biobank and Finnish cohorts were incorporated, for discovery and external validation. Meta-analysis was employed to integrate the results from both discovery and validation cohorts, thereby ensuring the reliability of findings.

**Results:**

ABCC8/KCNJ11 were associated with pancreatic cancer risk in both two-sample MR (odds ratio (OR): 15.058, per standard deviation unit (SD) change of glucose-lowering durg target perturbation equivalent to 1 SD unit of HbA_1c_ lowering; 95% confidence interval (95% CI): 3.824–59.295; *P-value* = 0.0001) and SMR (OR: 1.142; 95% CI: 1.013–1.287; *P-value* = 0.030) analyses. The mediation effect of body mass index (OR: 0.938; 95% CI: 0.884–0.995; proportion of mediation effect: 3.001%; *P-value* = 0.033) on ABCC8/KCNJ11 and pancreatic cancer was uncovered. Strong connections of DPP4 with anal carcinoma (OR: 0.123; 95% CI: 0.020–0.745; *P-value* = 0.023) and ICC (OR: 7.733; 95% CI: 1.743–34.310; *P-value* = 0.007) were detected. PPARG was associated with anal carcinoma (OR: 12.909; 95% CI: 3.217–51.795; *P-value* = 0.0003), HCC (OR: 36.507; 95% CI: 8.929-149.259; *P-value* < 0.0001), and pancreatic cancer (OR: 0.110; 95% CI: 0.071–0.172; *P-value* < 0.0001). SLC5A2 was connected with pancreatic cancer (OR: 8.096; 95% CI: 3.476–18.857; *P-value* < 0.0001). Weak evidence indicated the connections of GLP1R, GPD2, and PRKAB1 with anal carcinoma, cardia cancer, ICC, and rectum cancer. In addition, the corresponding results were consistently validated in both the validation cohorts and the integrated outcomes.

**Conclusions:**

Some glucose-lowering drugs were associated with gastrointestinal cancer risk, which might provide new ideas for gastrointestinal cancer treatment.

**Supplementary Information:**

The online version contains supplementary material available at 10.1186/s13578-024-01214-8.

## Introduction

Type 2 diabetes mellitus (T2DM), a chronic metabolic disease characterized by insulin resistance and elevated blood glucose levels, has become a pervasive global epidemic and is projected to impact 463 million adults in 2019 [1]. Moreover, the prevalence of this condition is escalating rapidly, with estimates suggesting that it will affect a staggering 700 million individuals by 2045 [1]. Achieving and maintaining optimal glycemic control necessitates the long-term administration of various glucose-lowering drugs for individuals with T2DM, including thiazolidinediones, dipeptidyl peptidase IV inhibitors, glucagon-like peptide-1 analogues, insulin/insulin analogues, sodium-glucose cotransporter inhibit, sulfonylureas, and metformin [2, 3].

Gastrointestinal cancers, including liver cancer, pancreatic cancer, gastric cancer, esophageal cancer, and colorectal cancer, stand as the leading contributors to cancer-related fatalities on a global scale [4]. T2DM, in turn, is recognized as a significant risk factor for various forms of gastrointestinal cancer, encompassing gastric cancer, hepatocellular carcinoma (HCC), and colorectal cancer [5–7]. However, the development of efficacious drugs with minimal side effects for alleviating gastrointestinal cancer symptoms has been limited [8]. A recent study unveiled a correlation between the utilization of certain glucose-lowering medications and the onset and progression of specific cancer types [9]. Metformin, a widely employed glucose-lowering drug in managing T2DM, exhibits substantial benefits concerning overall cancer incidence [10]. The use of metformin is significantly linked to reduced risks of cancer mortality, overall cancer incidence, and the incidence of liver cancer, colon cancer, rectal cancer, pancreatic cancer, gastric cancer, and esophageal cancer [6, 11]. Thiazolidinediones have also exhibited inhibitory effects on cancer cell growth [12, 13]. In contrast, the associations between other glucose-lowering drugs, such as alpha-glucosidase inhibitors, glucagon-like peptide-1 agonists, and dipeptidyl peptidase-4 inhibitors, and cancer risk remain inconclusive, likely influenced by specific medications, dosages, and treatment durations [9].

Mendelian randomization (MR) analysis, often referred to as a “natural” randomized controlled trial, holds the potential to mitigate confounding factors and elucidate causal relationships by leveraging genetic variations as instrumental variables (IV) [14]. Concurrently, MR analysis focused on drug targets serves as a widely adopted approach for investigating the causal effects of these targets on disease endpoints [15]. Given that cancer development is a protracted process, the utilization of drug target MR analysis becomes valuable in assessing the feasibility of drug repurposing and predicting potential side effects [16]. Notably, prior MR investigations have revealed associations between glucose-lowering drug targets and various conditions, including Alzheimer’s disease, non-alcoholic fatty liver disease, breast cancer, and prostate cancer, underscoring the significance of glucose-lowering drugs in the realm of disease management [17–19]. Some earlier MR studies suggested that specific targets related to metformin might protect against cancer and improve longevity [20, 21]. Time-related biases had been detected in observational studies of drug effects like metformin, which manifested the importance of using drug target MR analysis to reassess the potential effects of drug-lowering targets on gastrointestinal cancer risk before embarking on further long and expensive trials [22].

To date, a comprehensive MR study that delves into the causal relationship between glucose-lowering drug targets and the risk of gastrointestinal cancer remains scarce. So far as we know, only Yarmolinsky et al. examined the causal association between glucose-lowering drug targets and colon cancer [23]. Consequently, in this study we undertook a two-sample MR and summary-data-based Mendelian randomization (SMR) analysis to explore the potential associations between ten glucose-lowering drug targets and risks of seven types of gastrointestinal cancer, including anal cancer, cardiac cancer, gastric cancer, hepatocellular carcinoma, intrahepatic cholangiocarcinoma, pancreatic cancer, and rectal cancer. Moreover, we employed mediation MR analysis and colocalization analysis to provide additional insights and enhance our understanding of these causal relationships.

## Methods

### Study design

**Fig. 1:**
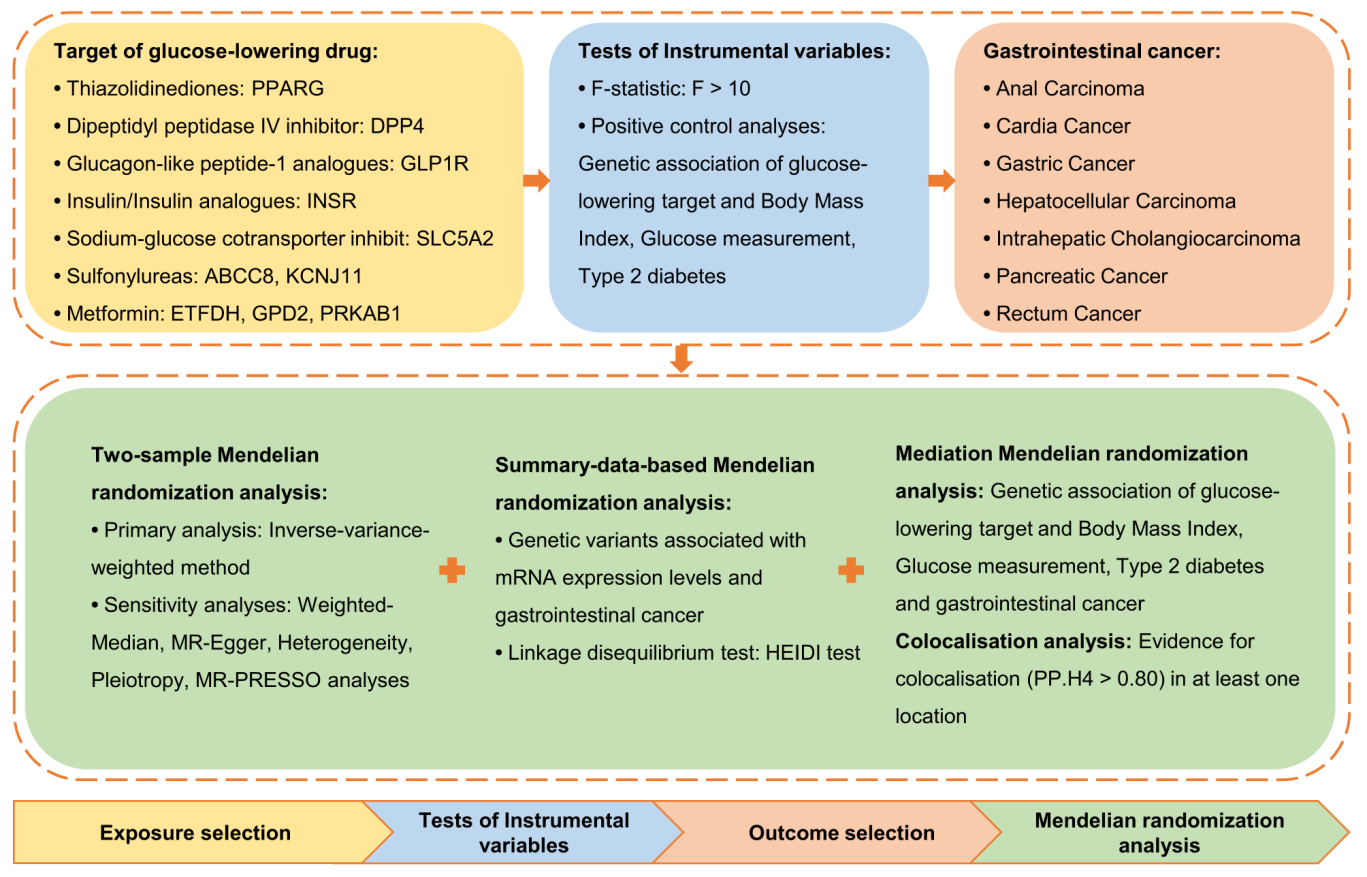
The flowchart of this study. *Abbreviations* HEIDI: heterogeneity in dependent instruments; MR-PRESSO: Mendelian Randomization Pleiotropy RESidual Sum and Outlier

The current study was reported according to the STROBE-MR statement [24]. Two-sample MR and SMR analyses were applied to reveal genetic relationships between glucose-lowering drug targets and gastrointestinal cancer risks. The validation of MR analyses’ causal estimation relied on three crucial assumptions: (i) the genetic IVs were closely connected with ten glucose-lowering drug targets; (ii) the independence of genetic IVs from confounding factors; and (iii) no direct effects of genetic IVs on gastrointestinal cancer risk other than through the glucose-lowering drug targets [25]. The random-effect inverse-variance weighted (IVW) method was the primary approach to elucidate the association between glucose-lowering drug targets and gastrointestinal cancer risks in the two-sample MR analyses [26]. We have also integrated validation cohorts for external corroboration of the pertinent results and employed meta-analysis to synthesize the findings from discovery and validation cohorts, guaranteeing the dependability and universality of our research outcomes. Similarly, we utilized a tool (https://shiny.cnsgenomics.com/mRnd/ ) to independently calculate the statistical power of the related analyses in both the discovery and validation cohorts, thereby assuring the efficacy of the analyses. Meanwhile, we conducted SMR analysis, which leverages expression quantitative trait loci (eQTLs) as instruments, to explore and validate the causal relationships between exposures and outcomes at the gene expression level [15]. Additionally, colocalization analysis, employing Bayes factor computation, was performed within a window of ± 500 KB around the gene encoding of each independent glucose-lowering drug target to calculate the posterior probabilities of connection between exposures and outcomes [27]. Furthermore, the mediation effects of some confounding factors (including body mass index (BMI), glucose measurement, and T2DM) and gastrointestinal cancer risks were uncovered through the two-step MR analyses to examine whether the observed relationship was direct. Figure 1 shows the detailed process of this study.

All summary data utilized in this study had been approved by the relevant institutional review board of each country on the basis of the Declaration of Helsinki, and all participants involved in these studies had signed the informed consent forms. Separate ethical approval was not required for this study.

### Extraction and selection of instrumental variables

Ten targets of seven different glucose-lowering drugs were eventually involved. Thiazolidinediones (TZDs) target PPARG (peroxisome proliferator-activated receptor gamma) precisely, activating it to increase tissue sensitivity to insulin and effectively lower blood sugar levels [28]. Dipeptidyl Peptidase IV Inhibitors, also known as DPP4 inhibitors, act on DPP4, reducing its activity to prevent the breakdown of incretins like GLP-1 [29]. This increase in GLP-1 levels encourages insulin secretion from pancreatic β-cells, helping to reduce blood sugar. Similarly, Glucagon-like Peptide-1 Analogues focus on GLP1R, boosting insulin secretion, hindering glycogenolysis, slowing gastric emptying, and increasing satiety [30]. Concurrently, Insulin and Insulin Analogues, which target INSR, mimic the effects of natural insulin, facilitating glucose uptake and utilization, thereby decreasing blood glucose levels [31]. Sodium-Glucose Cotransporter Inhibitors, or SGLT2 inhibitors, target SLC5A2 and block SGLT2 in the renal tubules, reducing glucose reabsorption and increasing urinary glucose excretion, leading to lower blood glucose levels [32]. Sulfonylureas, targeting ABCC8 and KCNJ11, close ATP-sensitive potassium channels, causing an increase in intracellular calcium and subsequent insulin release from β-cells [33]. Finally, Metformin acts on ETFDH, GPD2, and PRKAB1, activating AMPK, which reduces hepatic glycogenolysis and gluconeogenesis and enhances insulin sensitivity, collectively contributing to the reduction of blood glucose levels [34]. Each of these medications plays a unique and interconnected role in managing blood sugar levels through various mechanisms. Corresponding coding gene targets and pharmacologically active protein targets were searched from ChEMBL (https://www.ebi.ac.uk/chembl) and DrugBank (https://www.drugbank.ca) databases (Supplementary Table S1) [35, 36]. However, we also recognize that other significant genetic variations apart from these known targets might be related to glucose metabolism. To comprehensively solve this problem, we adopted a two-step strategy: (i) broad screening: during the GWAS data selection process, we employed an unbiased strategy to identify all potential genetic variations related to glucose metabolism. (ii) in-depth analysis: for those variations that showed a significant correlation with glucose metabolism in the preliminary screening, we conducted a more comprehensive and in-depth research search and review to ascertain whether they had been reported in previous studies and the potential biological roles they might play in disease [28–34]. Furthermore, we conducted a comprehensive search of existing GWAS databases. After excluding glucose-lowering drug targets for which adequate data could not be obtained, we incorporated ten targets of seven glucose-lowering drugs to the maximum extent possible, ensuring the comprehensiveness of our study.

For obtaining more effective IVs, the glucose-lowering drug targets were proxied by IVs collected from the summary genetic association data of HbA_1c_ measurement (*N* = 389,889) in the two-sample MR analyses (Supplementary Table S2) [37]. IVs were constructed within a range of ± 500 KB around the gene encodings for each of the ten glucose-lowering drug targets based on a significance threshold of *P-value* < 5 × 10^− 8^. Any IVs in high linkage disequilibrium (LD) with each other (*r*^*2*^ ≥ 0.01) were removed. Genetically proxied perturbation of per standard deviation unit (SD) change in glucose-lowering drug targets were scaled to represent an SD unit of HbA1c reduction.

In SMR analyses, the common (minor allele frequency (MAF) > 1%) eQTLs were identified from eQTLGen Consortium (https://www.eqtlgen.org/) and GTEx Consortium Version 8.0 (https://gtexportal.org/) as significant (*P-value* < 5 × 10^− 8^) IVs associated with the expression of DPP4, GPD2, ETFDH, GLP1R, INSR, KCNJ11, PPARG, PRKAB1, and SLC5A2 in blood tissue. Since no significant eQTLs about ABCC8 were found in blood tissue, we collected IVs related to the expression of ABCC8, specifically in muscle and skeletal tissues. Low weak linkage disequilibrium (*r*^*2*^ < 0.01) IVs were selected within the ± 500 KB windows of gene encodings for proxying glucose-lowering drug targets to ensure the high strength of the instruments.

### Validation of instrumental variables

The *F-statistic* method was hired to avoid potential weak IV bias with the criterion of *F-value* > 10 [38]. T2DM is the original indication of glucose-lowering drugs, and these drugs will ultimately affect the patient’s blood sugar levels [39]. A former meta-analysis indicated that some glucose-lowering drugs contribute to weight gains, such as sulfonylureas, insulin analogues, and thiazolidinediones, whereas GLP-1 analogues cause weight loss, which manifested body weight was another distinct phenotype affected by glucose-lowering drugs [40]. Therefore, BMI (*N* = 461,460), glucose measurement (*N* = 400,458), and T2DM (*N* = 655,666) selected from extensive summary data were utilized as positive controls to validate the strong association of IVs and genetically proxied glucose-lowering drug target perturbation [41, 42].

### Outcome data source for gastrointestinal cancer

Seven types of gastrointestinal cancer were selected from three large European ancestry databases (analyzed by Longda Jiang et al., Saori Sakaue et al., and Joshua D Backman et al. based on the UK Biobank and Finnland cohorts, respectively) as the discovery databases, including anal carcinoma (*N* = 456,348), cardia cancer (*N* = 456,348), gastric cancer (*N* = 456,348), HCC (*N* = 456,276), intrahepatic cholangiocarcinoma (ICC) (*N* = 456,348), pancreatic cancer (*N* = 635,945), and rectum cancer (*N* = 387,797) (Supplementary Table S2) [43–45]. In addition, for the purpose of validation, we have engaged additional cohorts from Finland (DF10, Public release: Dec 18, 2023) and other European populations (such as Kaiser Permanente Genetic Epidemiology Research on Adult Health and Aging and UK Biobank cohorts), as analyzed by Sara R Rashkin et al., Saori Sakaue et al., and Longda Jiang et al., respectively [43, 44, 46, 47]. These validation cohorts encompass anal carcinoma (*N* = 314,291), cardia cancer (*N* = 411,441), gastric cancer (*N* = 476,116), HCC (*N* = 314,693), ICC (*N* = 315,400), pancreatic cancer (*N* = 456,276), and rectal cancer (*N* = 316,683), with further details provided in Supplementary Table S2. Patients with gastrointestinal cancer were clinically or pathologically diagnosed based on the National Comprehensive Cancer Network standards. For no participants overlapping in the exposures and outcomes data, type I error was well avoided to ensure the strength of the MR analyses [48].

### Colocalization analysis

To scrutinize the alignment between the exposures and outcomes (specifically, the antihyperglycemic drug target and the susceptibility to gastrointestinal cancer) and to distinguish any confounding arising from linkage disequilibrium potentially ascribed to a shared causal variant, we employed colocalization analysis. This method leverages the computation of approximate Bayes factors to yield posterior probabilities, facilitating a more sophisticated interpretation of the interrelationships involved [27]. The colocalization analysis encompassed five hypotheses: (i) H0: neither the glucose-lowering drug targets nor gastrointestinal cancer possessed a causal variant within the genomic locus; (ii) H1: only the glucose-lowering drug targets harbored a causal variant; (iii) H2: only gastrointestinal cancer had a causal variant; (iv) H3: each of the glucose-lowering drug targets and gastrointestinal cancer had distinct causal variants; (v) H4: a shared causal variant was present for both glucose-lowering drug targets and gastrointestinal cancer [23]. To facilitate a thorough exploration of the genomic terrain encircling these critical regions, the colocalization analysis was executed by generating ± 500 kb windows surrounding the gene responsible for encoding each respective glucose-lowering drug target [27]. We utilized default parameters to conduct the colocalization, setting p1 = 1 × 10^− 4^ (the prior probability that a SNP is linked with the glucose-lowering drug target), p2 = 1 × 10^− 4^ (the prior probability that a SNP is linked with gastrointestinal cancer), and p12 = 1 × 10^− 5^ (the prior probability that a SNP is concurrently linked with both the glucose-lowering drug target and gastrointestinal cancer) [49]. A posterior probability for H4 (PP4) surpassing 0.8, under a variety of priors and windows, was construed as compelling evidence, signifying colocalization. The “coloc” (*Version 5.2.1*) and “LocusCompareR” (*Version 1.0.0*) packages were harnessed in the colocalization analysis to compute and graphically represent the outcomes.

### Sensitive analysis

In the two-sample MR analyses, the IVW method offers an unbiased estimate of causality, provided that all IVs are valid and free from pleiotropy [50]. To assess the heterogeneity of IVs and detect any pleiotropic effects, we conducted the Cochran’s Q test, with a significance threshold set at a *P-value* for heterogeneity < 0.05 [50]. As for potential horizontal pleiotropy, we performed additional analyses including weighted median and MR-Egger. the MR-Egger method allows for robust causal inference even when all IVs are potentially invalid and indicates the presence of unbalanced pleiotropy when the *P-value* for the intercept is < 0.05 [51]. Additionally, we applied the MR Pleiotropy RESidual Sum and Outlier (MR-PRESSO) global test to identify and adjust for outliers. Furthermore, in the SMR analysis, we employed the heterogeneity in dependent instruments (HEIDI) test, leveraging multiple SNPs within a genomic locus, to distinguish between associations of glucose-lowering drug targets with gastrointestinal cancer risk that are attributable to a shared genetic variant as opposed to genetic linkage [52]. A HEIDI test with a *P-value* > 0.01 indicated that the observed association between glucose-lowering drug targets and gastrointestinal cancer risk was not confounded by linkage disequilibrium [53].

### Statistical analysis

All analyses were performed using R software (*Version 4.2.3*) and SMR software (*Version 1.3.1*) [15]. R packages, including “TwoSampleMR” (*Version 0.5.6*) and “MR-PRESSO” (*Version 1.0*) were utilized. The Bonferroni-corrected significance level of *P-value* < 0.0007 (0.05/70, ten glucose-lowering drug targets and seven types of gastrointestinal cancer) was utilized to avert bias [54]. Associations with a *P-value* between 0.0007 and 0.05 was considered suggestive, while a *P*-*value* > 0.05 indicated no statistical association between glucose-lowering drug targets and gastrointestinal cancer.

## Results

### Positive control analysis

The *F-values* of each IV were calculated to avoid potential weak IV bias with the *F-values* > 10 (Supplementary Table S3). IVs utilized in two-sample MR and SMR analyses were tested through positive controls, including BMI, glucose measurement, and T2DM. Results of positive controls illustrated the credible association between IVs and BMI, glucose measurement, and T2DM indicating the effectiveness of IVs (Supplementary Table S4-S9).

### Two-sample MR analysis

**Fig. 2:**
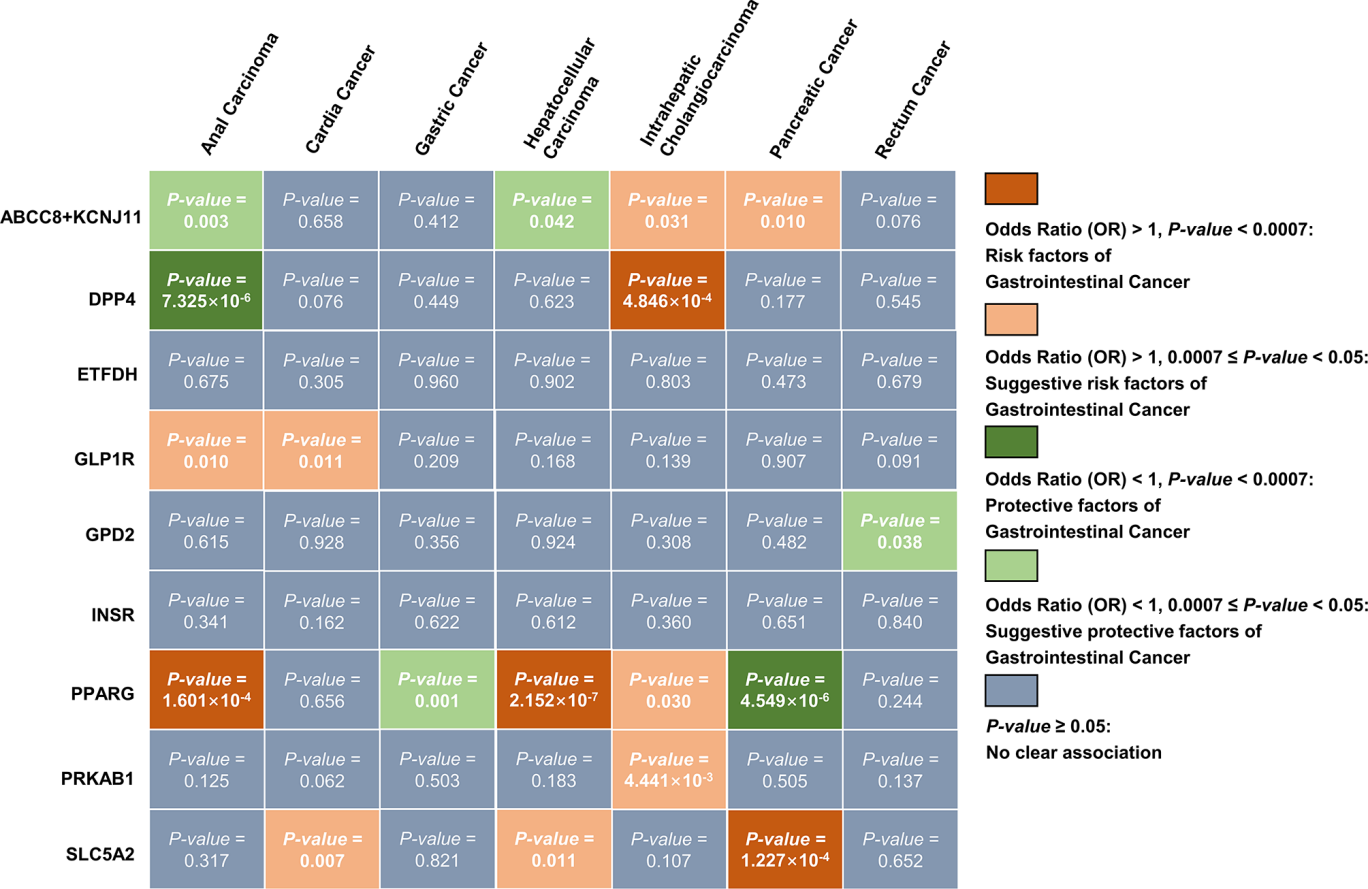
IVW method results of two-sample Mendelian randomization analyses on glucose-lowering drug target and risks of gastrointestinal cancer in the discovery databases. *Abbreviations* IVW method: Inverse-variance-weighted method

The results of two-sample MR analyses on glucose-lowering drug targets and gastrointestinal cancer risks are shown in Table 1; Fig. 2. The evidences of the meta-analyses of discovery and validation cohorts (*P*-value < 0.05, Table 1; Fig. 2) for the associations of ABCC8/KCNJ11 with anal carcinoma (odds ratio (OR) = 1.000 × 10^− 4^, per SD change of glucose-lowering drug target perturbation equivalent to 1 SD unit of HbA_1c_ lowering; 95% confidence interval (95% CI): 0.0001–0.013; *P-value* = 0.0002), HCC (OR = 0.003; 95% CI: 2.000 × 10^− 4^-0.028; *P-value* < 0.0001), but elevated risks of ICC (OR = 73.520; 95% CI: 10.312–524.150; *P-value* < 0.0001), pancreatic cancer (OR = 15.058; 95% CI: 3.824–59.295; *P-value* = 0.0001). The evidence underscored the substantial link of DPP4 with anal carcinoma (OR = 0.123; 95% CI: 0.020–0.745; *P-value* = 0.023) and ICC (OR = 7.733; 95% CI: 1.743–34.310; *P-value* = 0.007). However, no correlation was discerned between ETFDH, INSR, and gastrointestinal cancer risks. GLP1R was tentatively associated with anal carcinoma (OR = 49.160; 95% CI: 1.648–1.466 × 10^3^; *P-value* = 0.025) and cardia cancer (OR = 25.073; 95% CI: 5.064-124.149; *P-value* < 0.0001). GPD2 was associated with rectum cancer (OR = 0.031; 95% CI: 0.002–0.428; *P-value* = 0.010). PPARG were connected with anal carcinoma (OR = 12.909; 95% CI: 3.217–51.795; *P-value* = 0.0003), HCC (OR = 36.507; 95% CI: 8.929-149.259; *P-value* < 0.0001), and ICC (OR = 5.609; 95% CI: 2.772–11.347; *P-value* < 0.0001), but with lower risks of gastric cancer (OR = 0.691; 95% CI: 0.500-0.956; *P-value* = 0.026) and pancreatic cancer (OR = 0.110; 95% CI: 0.071–0.172; *P-value* < 0.0001). Moreover, PRKAB1 was associated with ICC (OR = 54.449; 95% CI: 3.434-863.226; *P-value* = 0.005). Furthermore, there were evidences uncovering the associations between SLC5A2 and cardia cancer (OR = 13.666; 95% CI: 4.610-40.515; *P-value* < 0.0001), HCC (OR = 29.465; 95% CI: 6.568-132.195; *P-value* < 0.0001), and pancreatic cancer (OR = 8.096; 95%CI: 3.476–18.857; *P-value* < 0.0001).

**Table 1:** Results of two-sample Mendelian randomization analyses on glucose-lowering drug targets and risks of gastrointestinal cancer

Gene	Cancer	Discovery Cohort		Validation Cohort		Combined	
		OR (95% CI)	P-value	OR (95% CI)	P-value	OR (95% CI)	P-value
ABCC8 + KCNJ11	Anal Carcinoma	2.749 × 10^− 4^ (1.18 × 10^− 6^, 0.064)	**0.003**	4.370 × 10^− 6^ (1.700 × 10^− 10^, 0.112)	**0.017**	1.000 × 10^− 4^ (0.0001, 0.013)	**0.0002**
	Cardia Cancer	4.217 (0.007, 2.46 × 10^3^)	0.658	0.518 (0.091, 2.952)	0.459	0.600 (0.112, 3.211)	0.550
	Gastric Cancer	0.107 (0.001, 22.430)	0.412	0.884 (0.364, 2.147)	0.786	0.835 (0.348, 2.005)	0.687
	Hepatocellular Carcinoma	0.009 (1.01 × 10^− 4^, 0.852)	**0.042**	0.001 (8.782 × 10^− 5^, 0.026)	**7.058 × 10** ^**− 6**^	0.003 (2.000 × 10^− 4^, 0.028)	**< 0.0001**
	Intrahepatic Cholangiocarcinoma	440.752 (1.751, 1.11 × 10^5^)	**0.031**	56.759 (6.941, 464.115)	**1.651 × 10** ^**− 4**^	73.520 (10.312, 524.150)	**< 0.0001**
	Pancreatic Cancer	8.440 (1.633, 43.635)	**0.010**	56.728 (4.720, 681.753)	**0.001**	15.058 (3.824, 59.295)	**0.0001**
	Rectum Cancer	3.805 (0.871, 16.620)	0.076	0.280 (0.073, 1.068)	0.062	0.911 (0.338, 2.457)	0.855
DPP4	Anal Carcinoma	4.453 × 10^− 6^ (2.04 × 10^− 8^, 0.001)	**7.325 × 10** ^**− 6**^	0.447 (0.020, 0.910)	**0.005**	0.123 (0.020, 0.745)	**0.023**
	Cardia Cancer	0.152 (0.004, 6.255)	0.321	5.092 (0.669, 38.723)	0.402	2.275 (0.383, 13.501)	0.366
	Gastric Cancer	0.192 (0.003, 13.660)	0.449	1.251 (0.430, 3.645)	0.681	1.120 (0.397, 3.160)	0.830
	Hepatocellular Carcinoma	19.221 (1.47 × 10^− 4^, 2.51 × 10^6^)	0.623	3.455 (0.312, 382.858)	0.389	3.988 (0.133, 120.054)	0.426
	Intrahepatic Cholangiocarcinoma	7.993 × 10^3^ (51.330, 1.25 × 10^6^)	**4.846 × 10** ^**− 4**^	3.991 (0.839, 18.977)	0.082	7.733 (1.743, 34.310)	**0.007**
	Pancreatic Cancer	0.315 (0.074, 1.334)	0.117	0.455 (0.053, 3.904)	0.472	0.353 (0.106, 1.170)	0.089
	Rectum Cancer	2.685 (0.110, 65.590)	0.545	8.268 (0.278, 246.197)	0.148	4.555 (0.445, 46.650)	0.202
ETFDH	Anal Carcinoma	0.004 (2.47 × 10^− 14^, 6.53 × 10^8^)	0.675	0.684 (0.025, 190.226)	0.990	0.589 (0.007, 48.429)	0.814
	Cardia Cancer	3.149 × 10^− 4^ (6.35 × 10^− 11^, 1.56 × 10^3^)	0.305	1.228 (0.129, 11.711)	0.953	1.032 (0.111, 9.616)	0.978
	Gastric Cancer	1.578 (3.17 × 10^− 8^, 7.86 × 10^7^)	0.96	0.666 (0.022, 20.340)	0.816	0.687 (0.024, 19.720)	0.826
	Hepatocellular Carcinoma	3.949 (1.34 × 10^− 9^, 1.16 × 10^10^)	0.902	4.262 (0.037, 485.767)	0.345	4.248 (0.042, 434.573)	0.540
	Intrahepatic Cholangiocarcinoma	0.070 (5.75 × 10^− 11^, 8.57 × 10^7^)	0.803	0.490 (0.000, 611.716)	0.845	0.401 (0.001, 341.417)	0.790
	Pancreatic Cancer	8.505 (0.025, 2.95 × 10^3^)	0.473	6.546 (0.142, 302.443)	0.132	7.082 (0.287, 174.724)	0.231
	Rectum Cancer	3.248 (0.012, 8.59 × 10^2^)	0.679	0.083 (0.001, 9.994)	0.309	0.394 (0.010, 14.895)	0.615
GLP1R	Anal Carcinoma	1.044 × 10^4^ (9.548, 1.14 × 10^7^)	**0.010**	9.431 (0.194, 458.377)	0.150	49.160 (1.648, 1.466 × 10^3^)	**0.025**
	Cardia Cancer	3.094 × 10^2^ (3.695, 2.59 × 10^4^)	**0.011**	17.191 (3.092, 95.583)	0.536	25.073 (5.064, 124.149)	**< 0.0001**
	Gastric Cancer	19.724 (0.189, 2.06 × 10^3^)	0.209	0.553 (0.293, 1.043)	0.067	1.526 (0.065, 35.899)	0.793
	Hepatocellular Carcinoma	0.046 (5.91 × 10^− 4^, 3.645)	0.168	2.666 (0.205, 34.673)	0.454	0.942 (0.103, 8.597)	0.958
	Intrahepatic Cholangiocarcinoma	63.028 (0.261, 1.52 × 10^4^)	0.139	0.975 (0.187, 5.091)	0.976	1.379 (0.283, 6.714)	0.690
	Pancreatic Cancer	1.085 (0.274, 4.292)	0.907	7.414 (0.732, 75.066)	0.090	1.792 (0.549, 5.846)	0.334
	Rectum Cancer	4.290 (0.795, 23.160)	0.091	1.136 (0.302, 4.273)	0.851	1.886 (0.665, 5.346)	0.233
GPD2	Anal Carcinoma	2.365 × 10^3^ (1.74 × 10^− 10^, 3.22 × 10^16^)	0.615	14.585 (0.004, 500.561)	0.380	17.511 (0.057, 5.406 × 10^3^)	0.328
	Cardia Cancer	0.509 (2.28 × 10^− 7^, 1.14 × 10^6^)	0.928	4.010 (0.516, 313.133)	0.078	3.648 (0.160, 83.440)	0.418
	Gastric Cancer	2.869 × 10^3^ (1.32 × 10^− 4^, 6.25 × 10^10^)	0.356	1.267 (0.040, 40.233)	0.893	1.729 (0.058, 51.164)	0.752
	Hepatocellular Carcinoma	2.161 (3.09 × 10^− 7^, 1.51 × 10^7^)	0.924	0.009 (7.779 × 10^− 8^, 1.132 × 10^3^)	0.434	0.065 (0.0001, 779.411)	0.568
	Intrahepatic Cholangiocarcinoma	3.333 × 10^4^ (6.74 × 10^− 5^, 1.65 × 10^13^)	0.308	0.002 (7.750 × 10^− 8^, 76.031)	0.254	0.078 (0.0001, 766.568)	0.586
	Pancreatic Cancer	8.064 (0.024, 2.71 × 10^3^)	0.482	12.495 (0.003, 4.623 × 10^4^)	0.547	9.334 (0.081, 1.076 × 10^3^)	0.357
	Rectum Cancer	0.004 (1.83 × 10^− 5^, 0.728)	**0.038**	0.062 (0.003, 1.277)	0.086	0.031 (0.002, 0.428)	**0.010**
INSR	Anal Carcinoma	1.095 × 10^3^ (0.001, 1.99 × 10^9^)	0.341	0.381 (0.008, 173.995)	0.885	0.889 (0.008, 97.817)	0.961
	Cardia Cancer	5.233 × 10^3^ (0.032, 8.60 × 10^8^)	0.162	9.896 (0.103, 94.798)	0.058	15.804 (0.594, 420.471)	0.099
	Gastric Cancer	0.031 (3.35 × 10^− 8^, 2.96 × 10^4^)	0.622	2.047 (0.139, 30.057)	0.601	1.756 (0.126, 24.528)	0.676
	Hepatocellular Carcinoma	0.050 (4.49 × 10^− 7^, 5.49 × 10^3^)	0.612	0.067 (2.086 × 10^− 4^, 21.519)	0.359	0.063 (4.000 × 10^− 4^, 11.094)	0.295
	Intrahepatic Cholangiocarcinoma	0.001 (4.81 × 10^− 10^, 2.41 × 10^3^)	0.360	0.036 (0.001, 1.513)	0.081	0.029 (0.001, 1.086)	0.056
	Pancreatic Cancer	0.397 (0.007, 21.720)	0.651	11.007 (0.023, 5.234 × 10^3^)	0.446	1.063 (0.037, 30.495)	0.972
	Rectum Cancer	0.669 (0.014, 33.000)	0.840	0.428 (0.032, 5.811)	0.524	0.492 (0.056, 4.292)	0.521
PPARG	Anal Carcinoma	42.852 (6.09, 301.523)	**1.601 × 10** ^**− 4**^	3.757 (0.519, 27.180)	0.190	12.909 (3.217, 51.795)	**0.0003**
	Cardia Cancer	1.440 (0.29, 7.151)	0.656	11.018 (5.995, 202.492)	0.109	3.851 (0.525, 28.257)	0.185
	Gastric Cancer	0.063 (0.012, 0.339)	**0.001**	0.758 (0.545, 1.055)	0.101	0.691 (0.500, 0.956)	**0.026**
	Hepatocellular Carcinoma	63.807 (13.260, 306.900)	**2.152 × 10** ^**− 7**^	3.644 (0.150, 88.367)	**0.026**	36.507 (8.929, 149.259)	**< 0.0001**
	Intrahepatic Cholangiocarcinoma	8.980 (1.241, 64.975)	**0.030**	5.238 (2.699, 12.193)	**0.046**	5.609 (2.772, 11.347)	**< 0.0001**
	Pancreatic Cancer	0.290 (0.171, 0.492)	**4.549 × 10** ^**− 6**^	0.010 (0.004, 0.023)	**2.371 × 10** ^**− 27**^	0.110 (0.071, 0.172)	**< 0.0001**
	Rectum Cancer	2.031 (0.616, 6.690)	0.244	1.470 (0.974, 22.190)	0.066	1.803 (0.699, 4.654)	0.223
PRKAB1	Anal Carcinoma	0.035 (4.917 × 10^− 4^, 2.539)	0.125	0.608 (7.671 × 10^− 6^, 482.378)	0.090	0.059 (0.001, 2.814)	0.151
	Cardia Cancer	20.741 (0.854, 503.700)	0.062	3.209 (0.805, 1.279 × 10^3^)	0.098	9.325 (0.836, 104.023)	0.070
	Gastric Cancer	0.285 (0.007, 11.250)	0.503	1.032 (0.551, 1.933)	0.922	0.995 (0.536, 1.847)	0.987
	Hepatocellular Carcinoma	0.097 (0.003, 3.010)	0.183	6.206 (0.541, 71.205)	0.143	1.541 (0.211, 11.276)	0.670
	Intrahepatic Cholangiocarcinoma	544.800 (7.099, 4.18 × 10^4^)	**4.441 × 10** ^**− 3**^	11.327 (0.369, 477.766)	0.067	54.449 (3.434, 863.226)	**0.005**
	Pancreatic Cancer	0.599 (0.133, 2.703)	0.505	0.157 (0.025, 0.975)	0.069	0.348 (0.109, 1.114)	0.075
	Rectum Cancer	2.403 (0.757, 7.626)	0.137	3.529 (0.110, 112.742)	0.333	2.497 (0.835, 7.470)	0.102
SLC5A2	Anal Carcinoma	0.153 (0.004, 6.058)	0.317	0.370 (0.009, 15.078)	0.599	0.237 (0.017, 3.230)	0.280
	Cardia Cancer	43.268 (2.771, 675.700)	**0.007**	11.038 (3.381, 36.036)	**6.951 × 10** ^**− 5**^	13.666 (4.610, 40.515)	**< 0.0001**
	Gastric Cancer	0.652 (0.016, 26.550)	0.821	29.569 (11.585, 551.493)	0.065	6.183 (0.156, 244.359)	0.332
	Hepatocellular Carcinoma	100.541 (2.926, 3.455 × 10^3^)	**0.011**	22.507 (4.289, 118.097)	0.092	29.465 (6.568, 132.195)	**< 0.0001**
	Intrahepatic Cholangiocarcinoma	46.219 (0.439, 4.868 × 10^3^)	0.107	1.165 (0.400, 3.393)	0.780	1.401 (0.494, 3.971)	0.527
	Pancreatic Cancer	6.650 (2.529, 17.486)	**1.227 × 10** ^**− 4**^	15.351 (2.686, 87.744)	**0.002**	8.096 (3.476, 18.857)	**< 0.0001**
	Rectum Cancer	1.284 (0.432, 3.814)	0.652	1.109 (0.525, 2.341)	0.786	1.162 (0.628, 2.152)	0.633

### SMR analysis

**Fig. 3:**
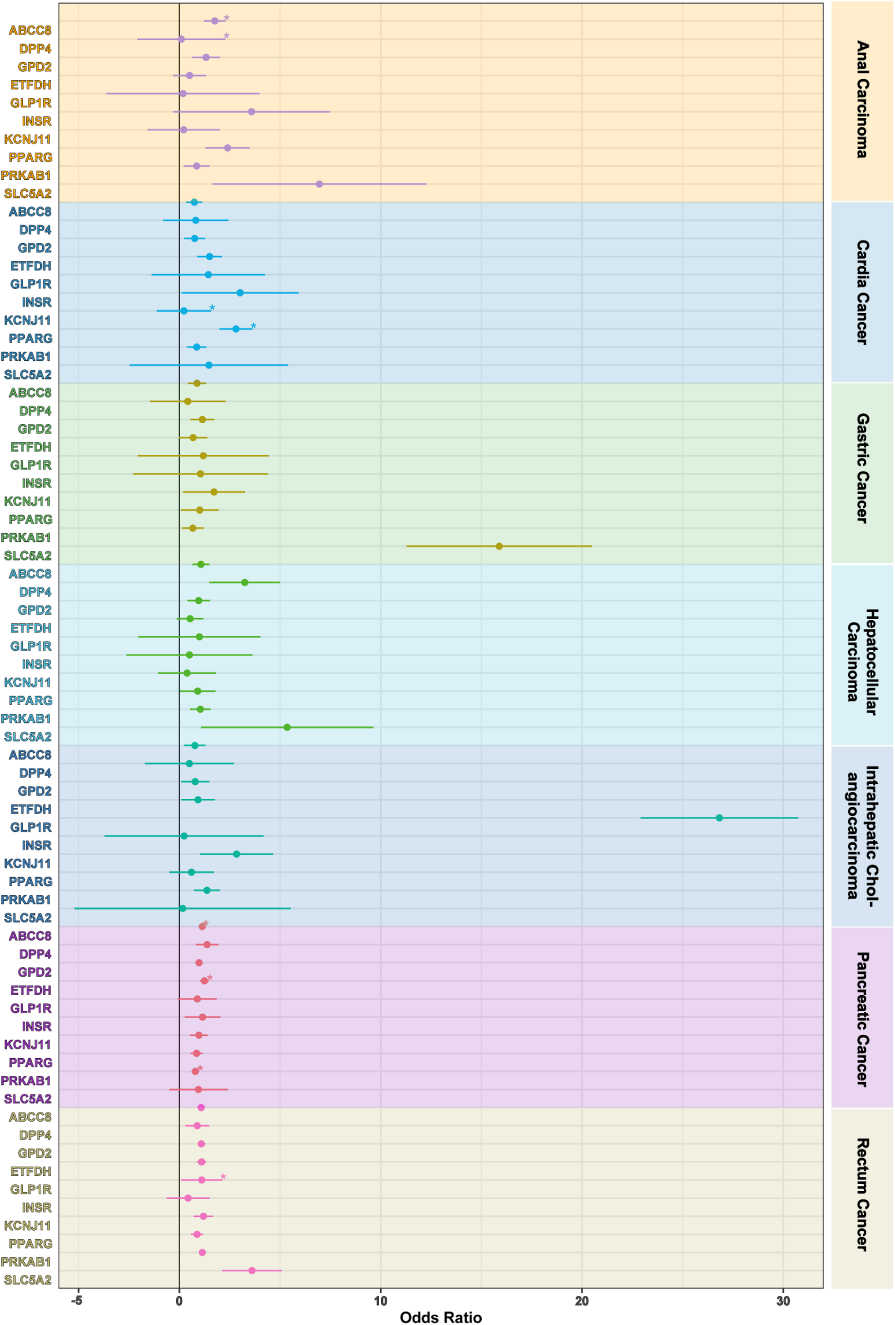
Results of summary-data-based Mendelian randomization analyses on glucose-lowering drug target and risks of gastrointestinal cancer

The results of SMR analyses on glucose-lowering drug targets and gastrointestinal cancer risks are illustrated in Fig. 3. The SMR analyses uncovered the genetic connections of anal carcinoma risk with ABCC8 (OR = 1.762; 95% CI: 1.034–3.022; *P-value* = 0.037) and DPP4 (OR = 0.100; 95% CI: 0.011–0.893; *P-value* = 0.039) (Supplementary Table S10). Risk of cardia cancer was connected with KCNJ11 (OR = 0.229; 95% CI: 0.059–0.884; *P-value* = 0.032) and PPARG (OR = 2.813; 95% CI: 1.239–6.388; *P-value* = 0.013) (Supplementary Table S11). However, no evidence manifested the associations of ten glucose-lowering drug targets with gastric cancer, HCC, ICC, and rectum cancer (Supplementary Table S12-S15). Moreover, ABCC8 (OR = 1.142; 95% CI: 1.013–1.287; *P-value* = 0.030), ETFDH (OR = 1.249; 95% CI: 1.006–1.550; *P-value* = 0.044), and PRKAB1 (OR = 0.798; 95% CI: 0.662–0.962; *P-value* = 0.018) were associated with pancreatic cancer risks (Supplementary Table S16).

### Mediation MR analysis

Two-step MR analyses were conducted to uncover the mediation effects of BMI, glucose measurement, and T2DM on glucose-lowering drug targets and gastrointestinal cancer risks, which had associations with two-sample MR analyses. The mediation effect of BMI on ABCC8/KCNJ11 and risk of pancreatic cancer are manifested in Table 2 (OR = 0.938; 95% CI: 0.884–0.995; proportion of mediation effect: 3.001%; *P-value* = 0.033). However, there were no mediation effects of BMI, glucose measurement, and T2DM on other glucose-lowering drug targets and gastrointestinal cancer risks (Supplementary Table S17-S18).

**Table 2:** The mediation effects of body mass index on glucose-lowering drug targets and risks of gastrointestinal cancer

Gene	Gastrointestinal Cancer	OR	95%CI	Proportion of mediation effect	P-Value
ABCC8 + KCNJ11	Anal Carcinoma	1.102	[0.757, 1.604]	1.185%	0.612
	Hepatocellular Carcinoma	0.908	[0.649, 1.269]	2.067%	0.571
	Intrahepatic Cholangiocarcinoma	0.759	[0.257, 2.244]	4.527%	0.618
	Pancreatic Cancer	0.938	[0.884, 0.995]	3.001%	**0.033**
DPP4	Anal Carcinoma	1.012	[0.694, 1.475]	0.097%	0.950
	Intrahepatic Cholangiocarcinoma	0.967	[0.327, 2.859]	0.377%	0.951
GLP1R	Anal Carcinoma	0.980	[0.672, 1.429]	0.218%	0.916
	Cardia Cancer	1.039	[0.609, 1.774]	0.669%	0.888
GPD2	Rectum Cancer	0.997	[0.958,1.038]	0.055%	0.880
PPARG	Anal Carcinoma	1.006	[0.690, 1.466]	0.156%	0.976
	Gastric Cancer	1.003	[0.845, 1.191]	0.113%	0.972
	Hepatocellular Carcinoma	0.994	[0.710, 1.393]	0.140%	0.973
	Intrahepatic Cholangiocarcinoma	0.984	[0.333, 2.908]	0.756%	0.976
	Pancreatic Cancer	0.996	[0.930, 1.067]	0.311%	0.913
PRKAB1	Intrahepatic Cholangiocarcinoma	1.381	[0.466, 4.091]	5.125%	0.560
SLC5A2	Cardia Cancer	1.222	[0.713, 2.092]	5.313%	0.466
	Hepatocellular Carcinoma	1.111	[0.789, 1.564]	2.277%	0.548
	Pancreatic Cancer	1.072	[0.978, 1.174]	3.663%	0.136

*Abbreviations* 95% CI: 95% confidence interval; OR: odds ratio

### Colocalization analysis

**Fig. 4:**
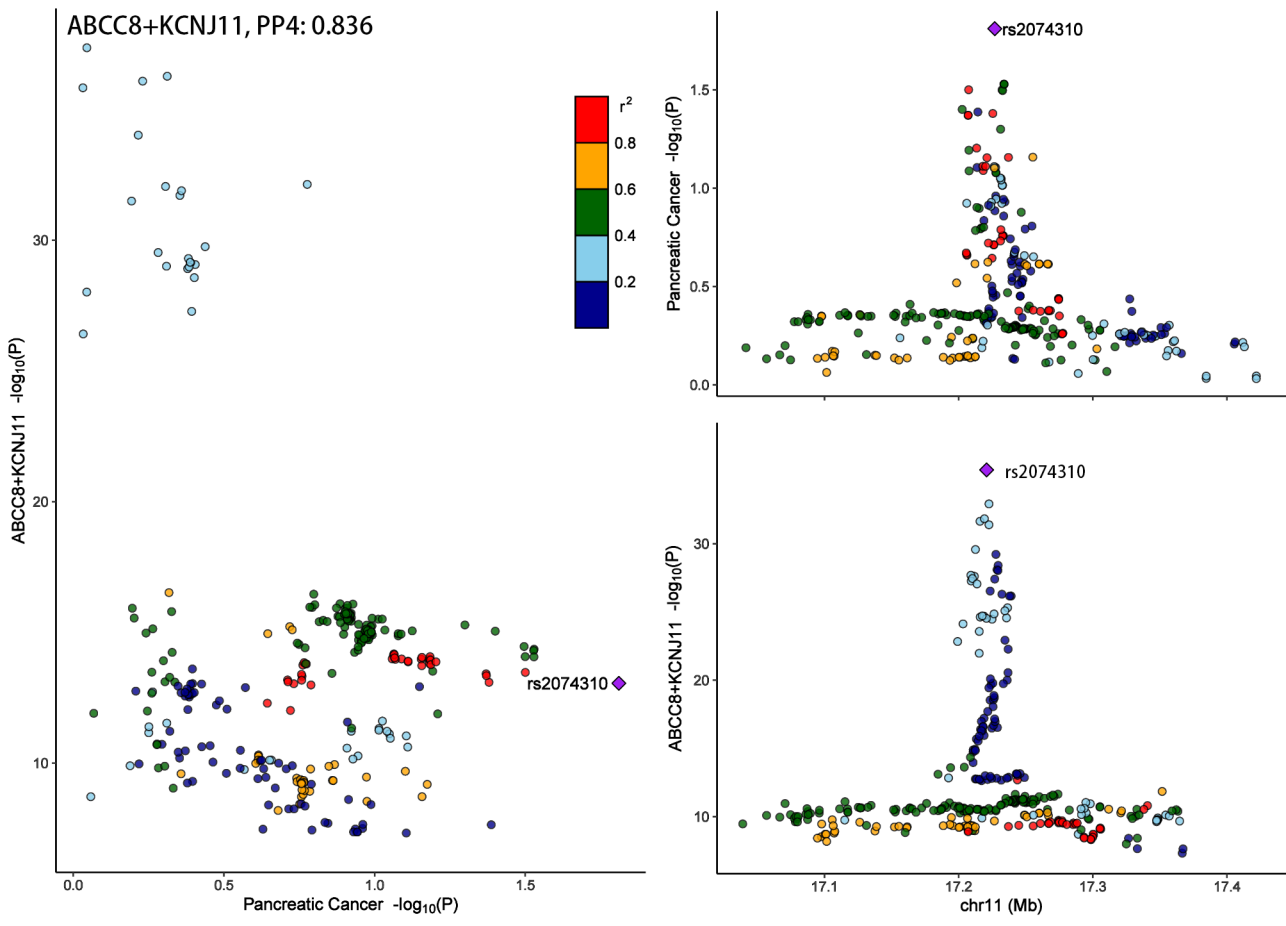
Regional Manhattan plot of associations of ABCC8 + KCNJ11 and risk of pancreatic cancer. The lead SNP is shown as a purple diamond. SNPs within ± 500 kb of the glucose-lowering drug target quantitative trait locus were included; p12 = 1 × 10^− 5^, prior probability a SNP is associated with both ABCC8 + KCNJ11 and pancreatic cancer

Colocalization analyses were performed to show the probability of sharing one common causal variant of glucose-lowering drug targets and gastrointestinal cancer risks (Supplementary Table S19). ABCC8/KCNJ11 were observed sharing gene regions with pancreatic cancer risk (PP4 = 0.836) (Fig. 4). Strong evidence suggested colocalization between DPP4, GLP1R, ABCC8/KCNJ11, and anal carcinoma (Supplementary Figure S1-S3). Meanwhile, ABCC8/KCNJ11 shared common gene regions with HCC (Supplementary Figure S4). Colocalizations between DPP4, ABCC8/KCNJ1, PRKAB1, and ICC were observed (Supplementary Figure S5-S7). Strong evidence uncovering the colocalization between the GPD2 and rectum cancer (Supplementary Figure S8).

### Sensitivity analysis and statistical power

The *P-values* of HEIDI tests in SMR analyses were all larger than 0.01, indicating horizontal pleiotropy due to linkage scenarios (Supplementary Table S10-S16). Additionally, the heterogeneity test, pleiotropy test, and MR-PRESSO test in two-sample MR analyses elucidated that no heterogeneity and pleiotropy existed with the *P-values* > 0.05 in both discovery and validation databases (Supplementary Table S20-S21). Furthermore, the statistical power of the related analyses in both the discovery and validation cohorts revealed that these analyses possess excellent efficacy (Supplementary Table S20-S21).

## Discussion

This study systematically investigated the causal relationship between ten glucose-lowering drug targets and the risks of seven types of gastrointestinal cancer. Associations of ABCC8/KCNJ11, DPP4, GLP1R, GPD2, PPARG, PRKAB1, and SLC5A2 with anal carcinoma, cardia cancer, gastric cancer, HCC, ICC, pancreatic cancer, and rectum cancer were revealed in two-sample MR analyses. In SMR analyses, ABCC8/KCNJ11, DPP4, PPARG, ETFDH, and PRKAB1 were associated with anal carcinoma, cardia cancer, and pancreatic cancer. Furthermore, ABCC8/KCNJ11, DPP4, GLP1R, PRKAB1, and GPD2 shared causal variants within anal cancer, HCC, ICC, pancreatic cancer, and rectum cancer according to the colocalization analyses. In addition, the mediation effect of BMI on the causal connection between ABCC8/KCNJ11 and pancreatic cancer risk was detected.

The pathogenesis of gastrointestinal cancer was characterized by a prolonged and intricate developmental cycle, with latent onset and convoluted mechanisms, which concomitantly posed challenges to the development of suitable therapeutic medications. Moreover, some glucose-lowering drugs were confirmed to be associated with cancer risk [9]. Some studies have demonstrated that metformin yielded tangible benefits in reducing the overall incidence of cancer, as well as decreasing the chances of developing colorectal cancer, esophageal cancer, liver cancer, and pancreatic cancer [55]. While metformin may have specific anticancer effects, these are not universally consistent. Therefore, in our study, we conducted a detailed investigation into the three drug targets of metformin (ETFDH, GPD2, and PRKAB1), and the discovery cohorts and integrated results revealed that GPD2 has an inhibitory effect on the occurrence of rectum cancer. Moreover, we found that PRKAB1 promotes the occurrence of ICC, while no association was found between ETFDH and the occurrence of gastrointestinal cancer. These findings suggest that metformin does not uniformly suppress all cancers. For clinical medication, further refining treatments in conjunction with the relevant drug targets would be prudent. Yang et al. uncovered that the use of gliclazide and glibenclamide was tied to a decline in cancer risk for T2D patients [56]. This was in harmony with our study where ABCC8/KCNJ11 was correlated with a higher risk of ICC and pancreatic cancer, as well as a lower risk of anal carcinoma and HCC. Simultaneously, the colocalization analyses revealed that ABCC8/KCNJ11 shared identical genetic regions with anal carcinoma, HCC, ICC, and pancreatic cancer. The promoting effect of ABCC8/KCNJ11 on pancreatic cancer was also observed in SMR analysis. Furthermore, BMI played a mediation role in the correlation between ABCC8/KCNJ11 and pancreatic cancer via the mediation MR analyses. Our mediation MR analyses indicated that glucose traits and T2DM had no causal effect on gastrointestinal cancer risks, which is consistent with earlier genetic studies detected by Motoki Iwasaki et al. and Neil Murphy et al. [57, 58]. These results suggest that in the clinical treatment of patients with type 2 diabetes concomitant with ICC/pancreatic cancer, the use of sulfonylureas should be avoided due to their potential role in promoting ICC and pancreatic cancer. DPP4 was associated with a lower risk of anal carcinoma in both two-sample MR analyses and SMR analyses. In comparison, it was associated with a higher risk of ICC in two-sample MR analyses. Interestingly, a recent meta-analysis found that DPP-4 inhibitors were not associated with an increased risk of pancreatic cancer, which was similar to our findings [59]. This finding implies that in a clinical setting, the use of dipeptidyl peptidase IV inhibitors to control blood sugar levels should be avoided in diabetic patients at risk for ICC due to the DPP4 potentially promoting the occurrence of ICC. Meantime, prior studies suggested that thiazolidinediones and glucagon-like peptide-1 analogues were connected with cancer risk, which could further validate our findings that PPARG and GLP1R were associated with increased anal carcinoma and cardia cancer risk [60]. This signifies that in a clinical setting, the use of thiazolidinediones and glucagon-like peptide-1 analogues should be avoided when treating diabetic patients who are at risk for anal carcinoma and cardia cancer. SLC5A2 was associated with increased risks of cardia cancer, HCC, and pancreatic cancer, which was in line with the findings of an earlier study by Tang et al. [61]. This suggests that caution should be exercised when using sodium-glucose cotransporter inhibitors to treat diabetic patients, as it may potentially elevate the risk of gastrointestinal cancer occurrence. While certain observational studies have hinted at a potential association between insulin usage and cancer susceptibility, these conclusions necessitate further refinement given the incomplete consideration of variables such as dosage, time span, and duration of insulin exposure [62]. This could tacitly bolster the discovery that INSR is not correlated with gastrointestinal malignancies, implying that insulin may not be linked with the risk of gastrointestinal cancer.

There are several strengths in this study. First, a comprehensive MR analysis based on two-sample MR and SMR analyses was undertaken to investigate the associations of ten glucose-lowering drug targets with the risk of seven types of gastrointestinal cancer. Second, the strength of selected IVs was validated through positive control analyses which effectively supported the suitability of these IVs as appropriate proxies. Third, mediation MR analyses and colocalization analyses were performed to explore the causalities of glucose-lowering drug targets and gastrointestinal cancer risks further. Fourth, the participants in this study were of European descent, which could minimize potential bias in population stratification.

Several limitations also exist in this study. Firstly, this study cannot investigate the relationship between the expression of ABCC8 in blood and the risk of gastrointestinal cancer, as there is no effective eQTL for ABCC8 in blood tissue. Secondly, this study only predicted the target effect of glucose-lowering drugs by including protein targets with sufficient records, whereas the off-target effect cannot be detected. Thirdly, this study was conducted in populations of European ancestry, raising concerns about the translating of these results to other races. It is critical to increase data collection in populations of non-European ancestry for target validation to inform drug development in general. Fourthly, gastrointestinal cancer incidence and evolution require a long cycle, and translating these genotype-phenotype associations into practical treatment strategies remains a significant challenge. Additionally, the database encompassing the gastrointestinal cancers implicated in this study lacks data about the staging and subtyping of the tumors, thereby precluding our ability to ascertain the specific stage of tumorigenesis at which the glucose-lowering drug targets exert their influence. This limitation may impact the generalizability of the study’s findings. Subsequent research endeavors could concentrate on tumor staging and typing to mitigate this limitation. Concurrently, some abnormal OR values were detected in our study. This may due to the assessment of gastrointestinal cancer risk in our study was based on changes in the target levels of each standard deviation glucose-lowering drug. While this approach takes into account the distribution of target levels within the study cohort, offering a standardized measure of the impact of fluctuations in target levels on risk, thus facilitating comparisons across different studies and populations, it is susceptible to significant variability within the standard deviation range of the studied glucose-lowering drug targets. Such variability could lead to observing anomalously high or low odds ratios in statistical analyses, associated with gastrointestinal cancer risk. In addition, the small fraction of positive outcomes in the employed database could also lead to statistical bias. Consequently, this issue merits further discussion with the advent of more comprehensive GWAS databases.

In summary, we have revealed associations between glucose-lowering drug targets and the risk of gastrointestinal cancer, which might provide new ideas for developing drugs for gastrointestinal cancer treatment. The underlying mechanisms should be elucidated in further research, and the role of glucose-lowering drug targets in gastrointestinal cancer risks could be evaluated in basic or clinical trials.

### Electronic supplementary material

Below is the link to the electronic supplementary material.

Supplementary Material 1: The details of supplementary tables S1-S21 and supplementary figures S1-S8

## Data Availability

All GWAS summary statistics data in this study are publicly available for download by qualified researchers.

## Abbreviations

95% CI: 95% confidence interval
BMI: body mass index
eQTL: expression quantitative trait loci
HCC: hepatocellular carcinoma
HEIDI test: heterogeneity in dependent instruments test
ICC: intrahepatic cholangiocarcinoma
IV: instrumental variable
MR: Mendelian randomization
MR-PRESSO: MR Pleiotropy RESidual Sum and Outlier
OR: odds ratio
SMR: summary-data-based Mendelian randomization
T2DM: type 2 diabetes mellitus
