# Supplementary material for: Association of glucose-lowering drug target and risk of gastrointestinal cancer: a mendelian randomization study
Source: Cell Biosci. 2024 Mar 19;14:36. doi: 10.1186/s13578-024-01214-8 (PMC10953268; doi:10.1186/s13578-024-01214-8)

**Supplementary materials**

**Supplementary Tables**

**Supplementary Table S1** | Information of glucose-lowering durg target utilized for selection of instrumental variables.

**Supplementary Table S2** | Information of exposure and outcome data used in the Mendelian randomization analyses. **Abbreviations:** eQTL: expression quantitative trait loci; GWAS: genome-wide association study.

**Supplementary Table S3** | Characteristics of SNPs extracted from exposure GWAS statistical summary data associated with glucose-lowering drug target. **Abbreviations:** GWAS: genome-wide association study; SE: standard error; SNP: single nucleotide polymorphisms.

**Supplementary Table S4** | Positive control analysis of SNPs involved in two-sample Mendelian rondomization analyses and body mass index. **Abbreviations:** 95% CI: 95% confidence interval; OR: odds ratio; SNP: single nucleotide polymorphisms.

**Supplementary Table S5** | Positive control analysis of SNPs involved in two-sample Mendelian rondomization analyses and glucose measurement. **Abbreviations:** 95% CI: 95% confidence interval; OR: odds ratio; SNP: single nucleotide polymorphisms.

**Supplementary Table S6** | Positive control analysis of SNPs involved in two-sample Mendelian rondomization analyses and type 2 diabetes. **Abbreviations:** 95% CI: 95% confidence interval; OR: odds ratio; SNP: single nucleotide polymorphisms.

**Supplementary Table S7** | Positive control analysis of eQTLs involved in summary-data-based Mendelian randomization analyses and body mass index. **Abbreviations:** 95% CI: 95% confidence interval; eQTL: expression quantitative trait loci; GWAS: genome-wide association study; HEIDI: heterogeneity in dependent instruments; OR: odds ratio; SE: standard error; SMR: summary-data-based Mendelian rondomization; SNP: single nucleotide polymorphisms.

**Supplementary Table S8** | Positive control analysis of eQTLs involved in summary-data-based Mendelian randomization analyses and glucose measurement. **Abbreviations:** 95% CI: 95% confidence interval; eQTL: expression quantitative trait loci; GWAS: genome-wide association study; HEIDI: heterogeneity in dependent instruments; OR: odds ratio; SE: standard error; SMR: summary-data-based Mendelian rondomization; SNP: single nucleotide polymorphisms.

**Supplementary Table S9** | Positive control analysis of eQTLs involved in summary-data-based Mendelian randomization analyses and type 2 diabetes. **Abbreviations:** 95% CI: 95% confidence interval; eQTL: expression quantitative trait loci; GWAS: genome-wide association study; HEIDI: heterogeneity in dependent instruments; OR: odds ratio; SE: standard error; SMR: summary-data-based Mendelian rondomization; SNP: single nucleotide polymorphisms.

**Supplementary Table S10** | SMR results of glucose-lowering drug target and anal carcinoma. **Abbreviations:** 95% CI: 95% confidence interval; eQTL: expression quantitative trait loci; GWAS: genome-wide association study; HEIDI: heterogeneity in dependent instruments; OR: odds ratio; SE: standard error; SMR: summary-data-based Mendelian rondomization; SNP: single nucleotide polymorphisms.

**Supplementary Table S11** | SMR results of glucose-lowering drug target and cardia cancer. **Abbreviations:** 95% CI: 95% confidence interval; eQTL: expression quantitative trait loci; GWAS: genome-wide association study; HEIDI: heterogeneity in dependent instruments; OR: odds ratio; SE: standard error; SMR: summary-data-based Mendelian rondomization; SNP: single nucleotide polymorphisms.

**Supplementary Table S12** | SMR results of glucose-lowering drug target and gastric cancer. **Abbreviations:** 95% CI: 95% confidence interval; eQTL: expression quantitative trait loci; GWAS: genome-wide association study; HEIDI: heterogeneity in dependent instruments; OR: odds ratio; SE: standard error; SMR: summary-data-based Mendelian rondomization; SNP: single nucleotide polymorphisms.

**Supplementary Table S13** | SMR results of glucose-lowering drug target and hepatocellular carcinoma. **Abbreviations:** 95% CI: 95% confidence interval; eQTL: expression quantitative trait loci; GWAS: genome-wide association study; HEIDI: heterogeneity in dependent instruments; OR: odds ratio; SE: standard error; SMR: summary-data-based Mendelian rondomization; SNP: single nucleotide polymorphisms.

**Supplementary Table S14** | SMR results of glucose-lowering drug target and intrahepatic cholangiocarcinoma. **Abbreviations:** 95% CI: 95% confidence interval; eQTL: expression quantitative trait loci; GWAS: genome-wide association study; HEIDI: heterogeneity in dependent instruments; OR: odds ratio; SE: standard error; SMR: summary-data-based Mendelian rondomization; SNP: single nucleotide polymorphisms.

**Supplementary Table S15** | SMR results of glucose-lowering drug target and rectum cancer. **Abbreviations:** 95% CI: 95% confidence interval; eQTL: expression quantitative trait loci; GWAS: genome-wide association study; HEIDI: heterogeneity in dependent instruments; OR: odds ratio; SE: standard error; SMR: summary-data-based Mendelian rondomization; SNP: single nucleotide polymorphisms.

**Supplementary Table S16** | SMR results of glucose-lowering drug target and pancreatic cancer. **Abbreviations:** 95% CI: 95% confidence interval; eQTL: expression quantitative trait loci; GWAS: genome-wide association study; HEIDI: heterogeneity in dependent instruments; OR: odds ratio; SE: standard error; SMR: summary-data-based Mendelian rondomization; SNP: single nucleotide polymorphisms.

**Supplementary Table S17 |** The mediation effects of glucose measurement on glucose-lowering drug target and risks of gastrointestinal cancer. **Abbreviations:** 95% CI: 95% confidence interval; OR: odds ratio.

**Supplementary Table S18 |** The mediation effects of type 2 diabetes on glucose-lowering drug target and risks of gastrointestinal cancer. **Abbreviations:** 95% CI: 95% confidence interval; OR: odds ratio.

**Supplementary Table S19 |** Colocalization analyses of glucose-lowering drug targets and gastrointestinal cancer risks.

**Supplementary Table S20 |** Sensitivity and power analyses of two-sample Mendelian randomization analyses in discovery cohorts. **Abbreviations:** SE: standard error; OR: odds ratio.

**Supplementary Table S21 |** Sensitivity and power analyses of two-sample Mendelian randomization analyses in validation cohorts. **Abbreviations:** SE: standard error; OR: odds ratio.

| **Supplementary Table S1** \| Information of glucose-lowering durg target utilized for selection of instrumental variables. | | | | | | |
| --- | --- | --- | --- | --- | --- | --- |
| **Drug** | **Drug Targets** | **Encoding Genes** | **Assembly** | **Probe ID** | **Gene chromosome** | **Gene Location** |
| Thiazolidinediones | Peroxisome proliferator-activated receptor gamma | PPARG | GRCh37.p13 | ENSG00000132170 | 3 | NC_000003.11 (12328867..12475843) |
| Dipeptidyl peptidase IV inhibitor | Dipeptidyl peptidase IV | DPP4 | GRCh37.p13 | ENSG00000197635 | 2 | NC_000002.11 (162848755..162930725) |
| Glucagon-like peptide-1 analogues | Glucagon-like peptide 1 receptor | GLP1R | GRCh37.p13 | ENSG00000112164 | 6 | NC_000006.11 (39016557..39059079) |
| Insulin/Insulin analogues | Insulin receptor | INSR | GRCh37.p13 | ENSG00000171105 | 19 | NC_000019.9 (7112276..7294425) |
| Sodium-glucose cotransporter inhibit | Sodium/glucose cotransporter 2 | SLC5A2 | GRCh37.p13 | ENSG00000140675 | 16 | NC_000016.9 (31494444..31502090) |
| Sulfonylureas | ATP-sensitive potassium channel | KCNJ11 | GRCh37.p13 | ENSG00000187486 | 11 | NC_000011.9 (17406795..17410893) |
|  | Peroxisome | ABCC8 | GRCh37.p13 | ENSG00000006071 | 11 | NC_000011.9 (17414045..17498392) |
| Metformin | Electron transfer flavoprotein-ubiquinone oxidoreductase | ETFDH | GRCh37.p13 | ENSG00000171503 | 4 | NC_000004.11 (159593448..159630775) |
|  | Mitochondrial glycerol-3-phosphate dehydrogenase | GPD2 | GRCh37.p13 | ENSG00000115159 | 2 | NC_000002.11 (157292064..157442915) |
|  | 5'-AMP-activated protein kinase subunit beta-1 | PRKAB1 | GRCh37.p13 | ENSG00000111725 | 12 | NC_000012.11 (120105938..120119424) |

| **Supplementary Table S2** \| Information of exposure and outcome data used in the Mendelian randomization analyses. | | | | | |
| --- | --- | --- | --- | --- | --- |
| **Characteristic** | **Resource/Author** | **Sample Size** | **Population** | **PubMed ID** | **Download Source** |
| **eQTL data** | | | | | |
| eQTL for DPP4, GPD2, ETFDH, GLP1R, INSR, KCNJ11, PPARG, PRKAB1 and SLC5A2 | eQTLGen Consortium | 31,684 | Predominantly European | 34475573 | [https://www.eqtlgen.org](https://www.eqtlgen.org/) |
| eQTL for ABCC8 | GTExV8 | 863 | Predominantly European | 29022597 | <https://www.gtexportal.org/home> |
| **GWAS summary data** | | | | | |
| HbA_1c_ measurement | Joelle Mbatchou et al. | 389,889 | European | 34017140 | <https://www.ebi.ac.uk/gwas/> |
| Body Mass Index | Ben Elsworth et al. | 461,460 | European | NA | <https://gwas.mrcieu.ac.uk/> |
| Glucose measurement | Alison R Barton et al. | 400,458 | European | 34226706 | <https://www.ebi.ac.uk/gwas/> |
| Type 2 diabetes | Angli Xue et al. | 655,666 | European | 30054458 | <https://gwas.mrcieu.ac.uk/> |
| **Discovery Cohort** | | | | | |
| Anal Carcinoma | Longda Jiang et al. | 456,348 | European | 34737426 | <https://www.ebi.ac.uk/gwas/> |
| Cardia Cancer | Longda Jiang et al. | 456,348 | European | 34737426 | <https://www.ebi.ac.uk/gwas/> |
| Gastric Cancer | Longda Jiang et al. | 456,348 | European | 34737426 | <https://www.ebi.ac.uk/gwas/> |
| Hepatocellular Carcinoma | Longda Jiang et al. | 456,276 | European | 34737426 | <https://www.ebi.ac.uk/gwas/> |
| Intrahepatic Cholangiocarcinoma | Longda Jiang et al. | 456,348 | European | 34737426 | <https://www.ebi.ac.uk/gwas/> |
| Pancreatic Cancer | Saori Sakaue et al. | 635,945 | Predominantly European | 34594039 | <https://www.ebi.ac.uk/gwas/> |
| Rectum Cancer | Joshua D Backman et al. | 387,797 | European | 34662886 | <https://www.ebi.ac.uk/gwas/> |
| **Verified Cohort** | | | | | |
| Anal Carcinoma | Mitja I Kurki et al. | 314,291 | European | 36653562 | <https://www.finngen.fi/en> |
| Cardia Cancer | Sara R Rashkin et al. | 411,441 | European | 32887889 | <https://www.ebi.ac.uk/gwas/> |
| Gastric Cancer | Saori Sakaue et al. | 476,116 | European | 34594039 | <https://www.ebi.ac.uk/gwas/> |
| Hepatocellular Carcinoma | Mitja I Kurki et al. | 314,693 | European | 36653562 | <https://www.finngen.fi/en> |
| Intrahepatic Cholangiocarcinoma | Mitja I Kurki et al. | 315,400 | European | 36653562 | <https://www.finngen.fi/en> |
| Pancreatic Cancer | Longda Jiang et al. | 456,276 | European | 34737426 | <https://www.ebi.ac.uk/gwas/> |
| Rectum Cancer | Mitja I Kurki et al. | 316,683 | European | 36653562 | <https://www.finngen.fi/en> |
| **Abbreviations:** eQTL: expression quantitative trait loci; GWAS: genome-wide association study. | | | | | |

| **Supplementary Table S3** \| Characteristics of SNPs extracted from exposure GWAS statistical summary data associated with glucose-lowering drug target. | | | | | | | | | |
| --- | --- | --- | --- | --- | --- | --- | --- | --- | --- |
| **Gene** | **SNP** | **Chromosome** | **Gene base pair** | **Effect allele** | **Other allele** | **Beta** | **SE** | **F-statistic** | **P-Value** |
| ABCC8+KCNJ11 | rs55683806 | 11 | 15016878 | A | T | 0.0194604 | 0.0042965 | 20.515 | 5.92E-09 |
| ABCC8+KCNJ11 | rs74643981 | 11 | 15082435 | G | T | 0.0432164 | 0.0064112 | 45.438 | 1.58E-11 |
| ABCC8+KCNJ11 | rs11023841 | 11 | 16078986 | C | G | 0.017452 | 0.0034504 | 25.583 | 4.24E-09 |
| ABCC8+KCNJ11 | rs11023870 | 11 | 16201396 | A | G | 0.0175197 | 0.0034492 | 25.800 | 3.79E-09 |
| ABCC8+KCNJ11 | rs2118359 | 11 | 16209846 | T | G | 0.0143639 | 0.0025112 | 32.719 | 1.07E-08 |
| ABCC8+KCNJ11 | rs1519126 | 11 | 16222976 | C | T | 0.0115576 | 0.0020151 | 32.897 | 9.71E-09 |
| ABCC8+KCNJ11 | rs10500829 | 11 | 16236176 | T | C | -0.0100276 | 0.0020421 | 24.112 | 9.09E-09 |
| ABCC8+KCNJ11 | rs11023883 | 11 | 16236447 | G | A | 0.0288904 | 0.0036725 | 61.884 | 3.64E-15 |
| ABCC8+KCNJ11 | rs77867718 | 11 | 16244538 | C | T | 0.0505824 | 0.0110501 | 20.954 | 4.70E-09 |
| ABCC8+KCNJ11 | rs4336994 | 11 | 16280948 | A | G | -0.0107377 | 0.0022247 | 23.295 | 1.39E-09 |
| ABCC8+KCNJ11 | rs117351529 | 11 | 16338009 | C | T | 0.0306141 | 0.0037019 | 68.389 | 1.34E-16 |
| ABCC8+KCNJ11 | rs7127006 | 11 | 16357680 | A | G | 0.0142755 | 0.0022561 | 40.036 | 2.49E-10 |
| ABCC8+KCNJ11 | rs11023928 | 11 | 16361573 | T | A | 0.0109625 | 0.0020854 | 27.635 | 1.47E-09 |
| ABCC8+KCNJ11 | rs12420958 | 11 | 17078885 | T | G | 0.0115033 | 0.0021678 | 28.157 | 1.12E-09 |
| ABCC8+KCNJ11 | rs664382 | 11 | 17184972 | T | A | 0.0120003 | 0.0021571 | 30.949 | 2.65E-08 |
| ABCC8+KCNJ11 | rs214086 | 11 | 17276919 | C | G | 0.01018 | 0.0020345 | 25.038 | 5.62E-09 |
| ABCC8+KCNJ11 | rs757081 | 11 | 17330136 | G | C | 0.0131901 | 0.0021368 | 38.104 | 6.71E-10 |
| ABCC8+KCNJ11 | rs61880293 | 11 | 17354951 | C | T | 0.020748 | 0.0037889 | 29.986 | 4.35E-08 |
| ABCC8+KCNJ11 | rs2883548 | 11 | 17356207 | G | A | -0.0145146 | 0.0021603 | 45.142 | 1.83E-11 |
| ABCC8+KCNJ11 | rs7110094 | 11 | 17379972 | A | G | -0.0140381 | 0.0030812 | 20.758 | 5.21E-09 |
| ABCC8+KCNJ11 | rs10734253 | 11 | 17385060 | G | C | -0.0138859 | 0.0020311 | 46.741 | 8.10E-12 |
| ABCC8+KCNJ11 | rs12288315 | 11 | 17396662 | C | G | 0.0144763 | 0.003127 | 21.431 | 3.67E-09 |
| ABCC8+KCNJ11 | rs757110 | 11 | 17396930 | A | C | -0.0219322 | 0.0021001 | 109.065 | 1.57E-25 |
| DPP4 | rs66524163 | 2 | 160408176 | C | T | -0.0135493 | 0.0021836 | 38.501 | 5.47E-10 |
| DPP4 | rs12692596 | 2 | 160409399 | T | C | 0.0127144 | 0.0020865 | 37.133 | 1.10E-09 |
| DPP4 | rs11679846 | 2 | 160470207 | C | T | 0.0122119 | 0.0023755 | 26.428 | 2.73E-09 |
| DPP4 | rs12053386 | 2 | 160494395 | C | T | -0.0137911 | 0.0020533 | 45.113 | 1.86E-11 |
| DPP4 | rs74774050 | 2 | 160509878 | A | G | 0.0151854 | 0.0031252 | 23.610 | 1.18E-09 |
| DPP4 | rs4420713 | 2 | 160569146 | C | G | 0.0101903 | 0.0021555 | 22.350 | 2.27E-09 |
| DPP4 | rs6738698 | 2 | 160577451 | C | G | -0.0099242 | 0.002238 | 19.664 | 9.23E-09 |
| DPP4 | rs79251743 | 2 | 160671076 | C | T | -0.0152401 | 0.0033481 | 20.720 | 5.32E-11 |
| DPP4 | rs76209259 | 2 | 160680109 | C | T | -0.0121607 | 0.0024201 | 25.249 | 5.04E-10 |
| DPP4 | rs2218909 | 2 | 160737882 | T | C | 0.0096338 | 0.0020294 | 22.536 | 2.06E-12 |
| DPP4 | rs113571356 | 2 | 160972031 | T | C | -0.0327059 | 0.0058605 | 31.145 | 2.39E-08 |
| DPP4 | rs78818053 | 2 | 161156692 | A | G | -0.0291628 | 0.0058775 | 24.619 | 6.99E-11 |
| DPP4 | rs561174039 | 2 | 161282458 | A | C | -0.0329425 | 0.0074254 | 19.682 | 9.15E-12 |
| DPP4 | rs150094097 | 2 | 161515741 | G | C | -0.0256006 | 0.0048244 | 28.159 | 1.12E-09 |
| DPP4 | rs77465985 | 2 | 161530385 | T | G | -0.0339888 | 0.0072639 | 21.894 | 2.88E-11 |
| DPP4 | rs77155259 | 2 | 161647730 | A | G | -0.0194317 | 0.0041302 | 22.136 | 2.54E-10 |
| DPP4 | rs75833670 | 2 | 161662764 | T | C | -0.0345593 | 0.0071628 | 23.279 | 1.40E-12 |
| DPP4 | rs34306396 | 2 | 161733401 | A | G | -0.0124741 | 0.0025202 | 24.499 | 7.44E-12 |
| DPP4 | rs150875074 | 2 | 161768645 | C | T | -0.0348138 | 0.0072525 | 23.042 | 1.58E-16 |
| DPP4 | rs77882688 | 2 | 161803542 | A | C | -0.0173995 | 0.0035683 | 23.777 | 1.08E-16 |
| DPP4 | rs75174596 | 2 | 161858727 | C | T | -0.0222429 | 0.0046183 | 23.197 | 1.46E-16 |
| DPP4 | rs76296250 | 2 | 161887315 | G | C | -0.009728 | 0.0021855 | 19.813 | 8.54E-16 |
| DPP4 | rs61748241 | 2 | 161894723 | A | G | -0.0323848 | 0.0072442 | 19.985 | 7.80E-12 |
| DPP4 | rs41268651 | 2 | 162017002 | C | T | -0.0332238 | 0.0073907 | 20.208 | 6.95E-11 |
| DPP4 | rs13389219 | 2 | 164672366 | T | C | -0.0150035 | 0.0020657 | 52.755 | 3.78E-13 |
| DPP4 | rs78970517 | 2 | 164697792 | T | C | -0.0162601 | 0.0031155 | 27.240 | 1.80E-17 |
| DPP4 | rs12692738 | 2 | 164701742 | C | T | -0.0116866 | 0.0024345 | 23.043 | 1.58E-11 |
| DPP4 | rs13017482 | 2 | 164706383 | C | T | -0.0098457 | 0.0020657 | 22.717 | 1.88E-12 |
| DPP4 | rs355838 | 2 | 164762653 | G | T | -0.0113776 | 0.0020774 | 29.996 | 4.33E-10 |
| DPP4 | rs12998590 | 2 | 164874210 | A | T | 0.009736 | 0.0020724 | 22.071 | 2.63E-08 |
| DPP4 | rs10202959 | 2 | 164919667 | C | T | 0.0114518 | 0.0023709 | 23.330 | 1.36E-08 |
| DPP4 | rs1975746 | 2 | 164938751 | A | G | 0.0106195 | 0.0020713 | 26.285 | 2.95E-08 |
| ETFDH | rs116311803 | 4 | 157491736 | G | A | -0.0158957 | 0.0034479 | 21.255 | 4.02E-08 |
| ETFDH | rs6843852 | 4 | 161211606 | T | C | 0.0092616 | 0.002012 | 21.189 | 4.16E-08 |
| GLP1R | rs72852340 | 6 | 36587027 | A | G | 0.0124752 | 0.0024229 | 26.511 | 2.62E-08 |
| GLP1R | rs2395655 | 6 | 36677919 | G | A | -0.0095454 | 0.0020792 | 21.076 | 4.41E-08 |
| GLP1R | rs9470964 | 6 | 39048320 | G | A | 0.0270651 | 0.0046939 | 33.247 | 8.12E-09 |
| GLP1R | rs10305420 | 6 | 39048860 | T | C | -0.0103526 | 0.002078 | 24.820 | 6.29E-10 |
| GLP1R | rs10305423 | 6 | 39049286 | T | C | -0.0290622 | 0.0057371 | 25.661 | 4.07E-08 |
| GLP1R | rs910167 | 6 | 39065355 | A | G | -0.027265 | 0.0055544 | 24.096 | 9.17E-11 |
| GLP1R | rs910166 | 6 | 39065392 | A | G | 0.0221889 | 0.003627 | 37.426 | 9.50E-10 |
| GLP1R | rs12214482 | 6 | 39068409 | G | A | 0.0149161 | 0.0027424 | 29.583 | 5.36E-12 |
| GLP1R | rs10305518 | 6 | 39087236 | G | T | 0.0277293 | 0.0043748 | 40.176 | 2.32E-10 |
| GLP1R | rs57143316 | 6 | 39101515 | C | T | 0.0252708 | 0.0047326 | 28.512 | 9.31E-10 |
| GLP1R | rs9462476 | 6 | 39103222 | G | A | -0.0109963 | 0.0021585 | 25.953 | 3.50E-09 |
| GLP1R | rs115366757 | 6 | 39138613 | T | C | 0.0206595 | 0.0045269 | 20.827 | 5.03E-09 |
| GLP1R | rs73731704 | 6 | 39152411 | A | G | 0.0195704 | 0.0043093 | 20.625 | 5.59E-09 |
| GLP1R | rs56384501 | 6 | 39153367 | A | G | 0.0112198 | 0.0021095 | 28.289 | 1.04E-08 |
| GLP1R | rs1109772 | 6 | 39321169 | A | G | 0.0268719 | 0.0059282 | 20.547 | 5.82E-0 |
| GLP1R | rs6904583 | 6 | 39390335 | T | C | 0.0368369 | 0.0063865 | 33.269 | 8.02E-09 |
| GLP1R | rs12176100 | 6 | 39394939 | T | C | 0.0176826 | 0.0036748 | 23.154 | 1.50E-09 |
| GLP1R | rs2504791 | 6 | 39870720 | T | C | 0.0091284 | 0.0020518 | 19.794 | 8.63E-09 |
| GLP1R | rs2984443 | 6 | 39909600 | T | C | 0.0109621 | 0.0021064 | 27.085 | 1.95E-09 |
| GLP1R | rs79142813 | 6 | 40996745 | T | C | -0.0346722 | 0.0063271 | 30.030 | 4.26E-08 |
| GLP1R | rs78088364 | 6 | 41115336 | T | A | -0.0322709 | 0.0062462 | 26.692 | 2.39E-09 |
| GLP1R | rs34375790 | 6 | 41174244 | A | G | -0.0175111 | 0.0035458 | 24.390 | 7.87E-09 |
| GLP1R | rs76599643 | 6 | 41257346 | G | A | -0.030786 | 0.0063653 | 23.392 | 1.32E-09 |
| GLP1R | rs10947980 | 6 | 41558001 | G | A | 0.012541 | 0.0022841 | 30.145 | 4.01E-08 |
| GPD2 | rs12615570 | 2 | 156573645 | G | A | 0.0106183 | 0.0022852 | 21.591 | 3.37E-11 |
| GPD2 | rs55920843 | 2 | 157556189 | G | T | -0.0456005 | 0.0092089 | 24.520 | 7.35E-12 |
| GPD2 | rs115087802 | 2 | 158028047 | A | T | -0.0191976 | 0.0043445 | 19.526 | 9.92E-11 |
| INSR | rs1043409 | 19 | 7471255 | T | A | 0.0191898 | 0.0043282 | 19.657 | 9.26E-10 |
| INSR | rs116937327 | 19 | 7474670 | A | G | 0.0146089 | 0.0032576 | 20.112 | 7.31E-10 |
| INSR | rs56238506 | 19 | 8227637 | G | C | 0.0120409 | 0.002351 | 26.230 | 3.03E-09 |
| INSR | rs112276586 | 19 | 8229417 | C | T | 0.0136074 | 0.0026588 | 26.194 | 3.09E-09 |
| INSR | rs7248003 | 19 | 8236164 | T | C | 0.012018 | 0.0022566 | 28.363 | 1.01E-09 |
| INSR | rs62119635 | 19 | 8266647 | G | T | -0.0115038 | 0.0024453 | 22.132 | 2.54E-10 |
| INSR | rs12974291 | 19 | 8353724 | G | A | -0.0110233 | 0.0023372 | 22.245 | 2.40E-09 |
| INSR | rs7249421 | 19 | 9604623 | C | G | 0.0118441 | 0.0025384 | 21.772 | 3.07E-12 |
| INSR | rs8102465 | 19 | 9720299 | T | C | 0.0119142 | 0.0025446 | 21.922 | 2.84E-11 |
| PPARG | rs13320580 | 3 | 11914707 | A | G | -0.0126085 | 0.0021146 | 35.552 | 2.48E-14 |
| PPARG | rs7631080 | 3 | 11936476 | A | T | -0.0128081 | 0.0023319 | 30.168 | 3.96E-08 |
| PPARG | rs116354045 | 3 | 11937657 | G | C | -0.0390164 | 0.0068155 | 32.772 | 1.04E-08 |
| PPARG | rs2600258 | 3 | 11963466 | G | A | -0.0142746 | 0.0024725 | 33.330 | 7.78E-09 |
| PPARG | rs17669026 | 3 | 12001361 | G | A | -0.0152739 | 0.0026792 | 32.500 | 1.19E-08 |
| PPARG | rs17669062 | 3 | 12002492 | C | T | -0.0261171 | 0.003757 | 48.326 | 3.61E-12 |
| PPARG | rs307606 | 3 | 12025626 | T | A | -0.0146743 | 0.0024999 | 34.457 | 4.36E-09 |
| PPARG | rs35976104 | 3 | 12048963 | A | G | -0.0152003 | 0.0023223 | 42.842 | 5.93E-11 |
| PPARG | rs307586 | 3 | 12068747 | T | C | -0.0288051 | 0.0031657 | 82.794 | 9.11E-20 |
| PPARG | rs550280052 | 3 | 12085385 | A | G | 0.0297091 | 0.0043848 | 45.906 | 1.24E-11 |
| PPARG | rs115633989 | 3 | 12134774 | G | A | -0.033201 | 0.0045809 | 52.529 | 4.24E-13 |
| PPARG | rs112110461 | 3 | 12145015 | G | A | -0.0430791 | 0.0054696 | 62.032 | 3.38E-15 |
| PPARG | rs310762 | 3 | 12182651 | C | T | 0.0229023 | 0.0020587 | 123.762 | 9.50E-29 |
| PPARG | rs78512510 | 3 | 12185289 | A | G | -0.0334192 | 0.0053715 | 38.707 | 4.92E-10 |
| PPARG | rs62240442 | 3 | 12186319 | A | G | 0.0277195 | 0.0039944 | 48.159 | 3.93E-12 |
| PPARG | rs79850813 | 3 | 12195709 | G | C | -0.0244328 | 0.0039484 | 38.292 | 6.09E-10 |
| PPARG | rs13089989 | 3 | 12217589 | A | T | -0.0315045 | 0.0023838 | 174.669 | 7.07E-40 |
| PPARG | rs310747 | 3 | 12218045 | G | T | -0.0255036 | 0.0020908 | 148.791 | 3.19E-34 |
| PPARG | rs13068313 | 3 | 12221461 | A | G | -0.0365457 | 0.0023013 | 252.187 | 8.66E-57 |
| PPARG | rs1822534 | 3 | 12225304 | G | A | -0.0407818 | 0.0020668 | 389.331 | 1.16E-86 |
| PPARG | rs17671592 | 3 | 12231914 | C | T | -0.0265086 | 0.0047932 | 30.586 | 3.19E-08 |
| PPARG | rs310750 | 3 | 12232174 | G | T | -0.0298808 | 0.0033273 | 80.652 | 2.69E-19 |
| PPARG | rs310748 | 3 | 12233339 | A | G | 0.0199156 | 0.0024467 | 66.254 | 3.96E-16 |
| PPARG | rs60192719 | 3 | 12233661 | G | A | 0.0290528 | 0.0025546 | 129.338 | 5.72E-30 |
| PPARG | rs7632481 | 3 | 12252467 | G | A | -0.0287148 | 0.00204 | 198.127 | 5.36E-45 |
| PPARG | rs17036143 | 3 | 12253228 | T | G | 0.0239252 | 0.0029829 | 64.333 | 1.05E-15 |
| PPARG | rs7641345 | 3 | 12255041 | G | A | -0.0183361 | 0.0030614 | 35.873 | 2.11E-09 |
| PPARG | rs112496224 | 3 | 12260416 | T | C | -0.0429422 | 0.0052331 | 67.336 | 2.29E-16 |
| PPARG | rs62242087 | 3 | 12260709 | T | C | 0.0250139 | 0.0038302 | 42.651 | 6.54E-11 |
| PPARG | rs2960420 | 3 | 12273013 | G | C | 0.0171524 | 0.0021204 | 65.433 | 6.01E-16 |
| PPARG | rs115381724 | 3 | 12274264 | G | A | -0.0380778 | 0.0066644 | 32.645 | 1.11E-08 |
| PPARG | rs2920499 | 3 | 12277559 | G | A | 0.0173555 | 0.0020273 | 73.286 | 1.12E-17 |
| PPARG | rs2920503 | 3 | 12282731 | T | C | 0.0148625 | 0.0022388 | 44.071 | 3.17E-11 |
| PPARG | rs17793951 | 3 | 12329238 | G | A | -0.0362662 | 0.00215 | 284.524 | 7.76E-64 |
| PPARG | rs568984015 | 3 | 12330303 | T | A | 0.0329643 | 0.0035727 | 85.134 | 2.79E-20 |
| PPARG | rs4518111 | 3 | 12335845 | C | A | -0.0348122 | 0.0020432 | 290.302 | 4.27E-65 |
| PPARG | rs2921186 | 3 | 12343970 | T | C | 0.0257436 | 0.0028654 | 80.716 | 2.61E-19 |
| PPARG | rs75262919 | 3 | 12377867 | T | C | -0.0241896 | 0.0036063 | 44.993 | 1.98E-11 |
| PPARG | rs895096 | 3 | 12396176 | A | C | 0.0148642 | 0.0022938 | 41.992 | 9.16E-11 |
| PPARG | rs2972163 | 3 | 12396523 | A | G | 0.0271794 | 0.0020161 | 181.742 | 2.02E-41 |
| PPARG | rs709154 | 3 | 12415335 | T | A | -0.0298435 | 0.0021171 | 198.718 | 3.98E-45 |
| PPARG | rs113303080 | 3 | 12423485 | A | G | -0.0410654 | 0.0056225 | 53.345 | 2.80E-13 |
| PPARG | rs73136795 | 3 | 12426911 | A | G | 0.0250607 | 0.0031716 | 62.435 | 2.75E-15 |
| PPARG | rs3105363 | 3 | 12429571 | G | A | 0.0264654 | 0.0024862 | 113.316 | 1.84E-26 |
| PPARG | rs1152002 | 3 | 12430372 | T | C | -0.0146744 | 0.0020564 | 50.921 | 9.62E-13 |
| PPARG | rs709159 | 3 | 12439704 | A | C | -0.0190191 | 0.0023675 | 64.538 | 9.47E-16 |
| PPARG | rs66603943 | 3 | 12443182 | C | T | -0.0185979 | 0.0025245 | 54.272 | 1.75E-13 |
| PPARG | rs4684104 | 3 | 12443350 | G | A | 0.0149211 | 0.0020532 | 52.815 | 3.67E-13 |
| PPARG | rs10440120 | 3 | 12445465 | A | C | -0.0176583 | 0.0027626 | 40.857 | 1.64E-10 |
| PPARG | rs1152008 | 3 | 12446113 | G | T | -0.0194585 | 0.0020739 | 88.032 | 6.44E-21 |
| PPARG | rs11716727 | 3 | 12447513 | T | C | 0.0148391 | 0.0022929 | 41.883 | 9.69E-11 |
| PPARG | rs71624937 | 3 | 12448369 | A | G | -0.0186003 | 0.0022528 | 68.172 | 1.50E-16 |
| PPARG | rs9872031 | 3 | 12454962 | A | G | 0.0238776 | 0.0020398 | 137.034 | 1.19E-31 |
| PPARG | rs58691354 | 3 | 12456095 | T | C | 0.0228661 | 0.0028031 | 66.543 | 3.42E-16 |
| PPARG | rs11917039 | 3 | 12465176 | C | G | -0.0192945 | 0.0027071 | 50.798 | 1.02E-12 |
| PPARG | rs6763925 | 3 | 12474103 | C | T | -0.0214435 | 0.002219 | 93.382 | 4.31E-22 |
| PPARG | rs2655261 | 3 | 12521138 | A | C | -0.0144459 | 0.0020213 | 51.077 | 8.88E-13 |
| PPARG | rs79185322 | 3 | 12539767 | A | C | -0.0235996 | 0.0035497 | 44.201 | 2.96E-11 |
| PPARG | rs12492608 | 3 | 12563748 | T | A | -0.0145453 | 0.0024835 | 34.303 | 4.72E-09 |
| PPARG | rs713178 | 3 | 12574485 | C | T | -0.0142715 | 0.0023258 | 37.654 | 8.45E-10 |
| PPARG | rs9855183 | 3 | 12576763 | T | C | -0.021458 | 0.0033665 | 40.627 | 1.84E-10 |
| PPARG | rs2454429 | 3 | 12579221 | A | G | -0.0161446 | 0.0020393 | 62.674 | 2.44E-15 |
| PPARG | rs73130305 | 3 | 12579576 | T | C | -0.0254889 | 0.0024389 | 109.223 | 1.45E-25 |
| PPARG | rs3729931 | 3 | 12585017 | A | G | -0.0215461 | 0.0020955 | 105.725 | 8.47E-25 |
| PPARG | rs5746223 | 3 | 12599926 | C | T | -0.0243184 | 0.0032719 | 55.241 | 1.07E-13 |
| PPARG | rs75926229 | 3 | 12670472 | C | A | -0.0146843 | 0.0021445 | 46.887 | 7.52E-12 |
| PPARG | rs6766304 | 3 | 12679305 | A | C | -0.0227921 | 0.0036208 | 39.624 | 3.08E-10 |
| PPARG | rs9874557 | 3 | 12679468 | G | A | -0.0149248 | 0.0023558 | 40.135 | 2.37E-10 |
| PPARG | rs9874315 | 3 | 12686463 | A | C | 0.0153726 | 0.0020369 | 56.959 | 4.45E-14 |
| PPARG | rs80266055 | 3 | 12700527 | A | G | -0.0252985 | 0.0033844 | 55.877 | 7.72E-14 |
| PPARG | rs2055740 | 3 | 12749510 | A | G | -0.0251026 | 0.004597 | 29.819 | 4.74E-08 |
| PPARG | rs112938164 | 3 | 12752468 | C | T | -0.0253649 | 0.004468 | 32.229 | 1.37E-08 |
| PPARG | rs6770851 | 3 | 12756064 | T | C | -0.0223173 | 0.0028844 | 59.864 | 1.02E-14 |
| PPARG | rs2305398 | 3 | 12815357 | G | A | -0.0153642 | 0.002074 | 54.878 | 1.28E-13 |
| PRKAB1 | rs34965774 | 12 | 117974568 | A | G | 0.0215739 | 0.0030068 | 51.481 | 7.23E-13 |
| PRKAB1 | rs2254036 | 12 | 118126177 | A | G | -0.0229859 | 0.0041923 | 30.062 | 4.18E-08 |
| PRKAB1 | rs795478 | 12 | 118180948 | G | A | -0.0199978 | 0.0021956 | 82.956 | 8.39E-20 |
| PRKAB1 | rs708863 | 12 | 118243895 | G | T | -0.015577 | 0.002021 | 59.410 | 1.28E-14 |
| PRKAB1 | rs461066 | 12 | 118281153 | G | T | 0.0207395 | 0.0026698 | 60.344 | 7.96E-15 |
| PRKAB1 | rs551546 | 12 | 118294383 | C | T | 0.0225439 | 0.0041 | 30.233 | 3.83E-08 |
| PRKAB1 | rs459547 | 12 | 118296289 | A | G | 0.0214896 | 0.002222 | 93.531 | 4.00E-22 |
| PRKAB1 | rs542689 | 12 | 118348856 | C | T | -0.0134289 | 0.0020467 | 43.049 | 5.34E-11 |
| PRKAB1 | rs73226260 | 12 | 120942738 | A | G | -0.0387016 | 0.0055863 | 47.996 | 4.27E-12 |
| PRKAB1 | rs10082850 | 12 | 121774117 | A | G | 0.0156542 | 0.0025994 | 36.268 | 1.72E-09 |
| PRKAB1 | rs11553699 | 12 | 121779004 | G | A | 0.0173644 | 0.0030623 | 32.153 | 1.42E-08 |
| PRKAB1 | rs1154514 | 12 | 121936982 | T | C | 0.0143369 | 0.0020117 | 50.790 | 1.03E-12 |
| PRKAB1 | rs12830351 | 12 | 122037833 | T | C | 0.0123316 | 0.0020487 | 36.230 | 1.75E-09 |
| PRKAB1 | rs11043299 | 12 | 122045233 | C | T | 0.0162416 | 0.0022341 | 52.851 | 3.60E-13 |
| PRKAB1 | rs1916334 | 12 | 122046388 | A | G | -0.0141903 | 0.0025501 | 30.965 | 2.63E-08 |
| PRKAB1 | rs7137049 | 12 | 122055478 | C | A | 0.0128561 | 0.0020183 | 40.574 | 1.89E-10 |
| PRKAB1 | rs4758689 | 12 | 122126457 | T | A | -0.0138894 | 0.002538 | 29.948 | 4.44E-08 |
| PRKAB1 | rs7487292 | 12 | 122129840 | G | T | 0.013627 | 0.0020406 | 44.596 | 2.42E-11 |
| PRKAB1 | rs7972875 | 12 | 122178228 | T | C | 0.0137921 | 0.0020167 | 46.773 | 7.97E-12 |
| PRKAB1 | rs11059508 | 12 | 122180775 | T | C | -0.015454 | 0.0025734 | 36.062 | 1.91E-09 |
| PRKAB1 | rs75810210 | 12 | 122319096 | T | C | -0.0236055 | 0.0035216 | 44.931 | 2.04E-11 |
| PRKAB1 | rs10998 | 12 | 122504865 | A | G | 0.0135491 | 0.0023162 | 34.218 | 4.93E-09 |
| PRKAB1 | rs147730268 | 12 | 122539929 | T | G | -0.0267951 | 0.0036278 | 54.553 | 1.51E-13 |
| PRKAB1 | rs6489157 | 12 | 122617126 | C | G | 0.0130046 | 0.0022724 | 32.752 | 1.05E-08 |
| SLC5A2 | rs3814877 | 16 | 30031356 | T | G | 0.0165207 | 0.0020503 | 64.930 | 7.76E-16 |
| SLC5A2 | rs7542 | 16 | 30114519 | C | G | -0.0116114 | 0.0020269 | 32.817 | 1.01E-08 |
| SLC5A2 | rs138447338 | 16 | 30502164 | G | A | 0.0209693 | 0.0038461 | 29.725 | 4.98E-08 |
| SLC5A2 | rs34114657 | 16 | 30573109 | C | T | 0.0232786 | 0.0024442 | 90.708 | 1.66E-21 |
| SLC5A2 | rs11647825 | 16 | 30601625 | T | A | 0.0141217 | 0.0025861 | 29.818 | 4.75E-08 |
| SLC5A2 | rs9927137 | 16 | 30622979 | G | A | 0.0156323 | 0.0020212 | 59.819 | 1.04E-14 |
| SLC5A2 | rs13338946 | 16 | 30689537 | C | T | 0.0231006 | 0.0023005 | 100.834 | 1.00E-23 |
| SLC5A2 | rs370871117 | 16 | 30749046 | C | G | -0.0158637 | 0.0021115 | 56.446 | 5.78E-14 |
| SLC5A2 | rs28436836 | 16 | 30749091 | G | C | -0.0160984 | 0.0022211 | 52.534 | 4.23E-13 |
| SLC5A2 | rs4889490 | 16 | 30811726 | T | G | -0.0142861 | 0.0020606 | 48.064 | 4.12E-12 |
| SLC5A2 | rs28421305 | 16 | 30826807 | A | G | 0.0232506 | 0.0022635 | 105.518 | 9.41E-25 |
| SLC5A2 | rs8044030 | 16 | 30916669 | A | G | -0.0127688 | 0.0020728 | 37.946 | 7.27E-10 |
| SLC5A2 | rs56039835 | 16 | 30918685 | C | T | -0.0126991 | 0.0022055 | 33.154 | 8.51E-09 |
| SLC5A2 | rs7200879 | 16 | 30936251 | G | A | 0.0229645 | 0.0023505 | 95.456 | 1.51E-22 |
| SLC5A2 | rs12443808 | 16 | 30985550 | G | C | -0.0155067 | 0.0020814 | 55.504 | 9.32E-14 |
| SLC5A2 | rs17855121 | 16 | 30992848 | C | T | 0.0154563 | 0.0023405 | 43.613 | 4.00E-11 |
| SLC5A2 | rs1108431 | 16 | 31043286 | T | C | 0.0123371 | 0.0020788 | 35.222 | 2.94E-09 |
| SLC5A2 | rs9923231 | 16 | 31096368 | T | C | -0.0116341 | 0.0020776 | 31.356 | 2.15E-08 |
| SLC5A2 | rs1060506 | 16 | 31122128 | T | C | 0.0167794 | 0.0022273 | 56.753 | 4.94E-14 |
| SLC5A2 | rs12929023 | 16 | 31369143 | A | G | 0.0161699 | 0.0028581 | 32.008 | 1.54E-08 |
| SLC5A2 | rs11150620 | 16 | 31380868 | C | G | -0.0139127 | 0.0022185 | 39.328 | 3.58E-10 |
| SLC5A2 | rs12932429 | 16 | 31391824 | C | T | -0.0283107 | 0.0046889 | 36.455 | 1.56E-09 |
| SLC5A2 | rs8050500 | 16 | 31393250 | C | T | -0.0261764 | 0.0020239 | 167.281 | 2.90E-38 |
| SLC5A2 | rs45625038 | 16 | 31407654 | T | C | 0.0392602 | 0.0059066 | 44.181 | 2.99E-11 |
| SLC5A2 | rs28675289 | 16 | 31451931 | T | C | -0.0384834 | 0.0049407 | 60.670 | 6.75E-15 |
| SLC5A2 | rs8057207 | 16 | 31513259 | T | C | -0.0124869 | 0.0020926 | 35.607 | 2.41E-09 |
| SLC5A2 | rs9929691 | 16 | 31540875 | T | C | 0.0169729 | 0.0028197 | 36.232 | 1.75E-09 |
| SLC5A2 | rs28692853 | 16 | 31561709 | A | C | -0.0141113 | 0.0020166 | 48.968 | 2.60E-12 |
| SLC5A2 | rs145785014 | 16 | 31579005 | T | C | -0.0281488 | 0.0045115 | 38.929 | 4.39E-10 |
| SLC5A2 | rs34148591 | 16 | 31653541 | T | C | -0.0130957 | 0.0022175 | 34.877 | 3.51E-09 |
| SLC5A2 | rs1232538 | 16 | 31822491 | T | G | 0.0137088 | 0.0022542 | 36.984 | 1.19E-09 |
| SLC5A2 | rs241916 | 16 | 33814274 | T | C | -0.0243781 | 0.0044274 | 30.318 | 3.67E-08 |
| **Abbreviations:** GWAS: genome-wide association study; SE: standard error; SNP: single nucleotide polymorphisms. | | | | | | | | | |

| **Supplementary Table S4** \| Positive control analysis of SNPs involved in two-sample Mendelian rondomization analyses and body mass index. | | | |
| --- | --- | --- | --- |
| **Gene** | **OR** | **95%CI** | **P-value** |
| ABCC8+KCNJ11 | 0.743 | [0.658, 0.839] | **1.61E-06** |
| DPP4 | 0.964 | [0.764, 1.217] | 0.759 |
| ETFDH | 1.936 | [1.127, 3.326] | **0.017** |
| GLP1R | 1.064 | [0.933, 1.212] | 0.357 |
| GPD2 | 0.909 | [0.607, 1.363] | 0.644 |
| INSR | 1.065 | [0.820, 1.383] | 0.638 |
| PPARG | 0.982 | [0.928, 1.040] | 0.542 |
| PRKAB1 | 1.416 | [1.187, 1.688] | **1.067E-04** |
| SLC5A2 | 1.379 | [1.145, 1.662] | **7.193E-04** |
| **Abbreviations:** 95% CI: 95% confidence interval; OR: odds ratio; SNP: single nucleotide polymorphisms. | | | |

| **Supplementary Table S5** \| Positive control analysis of SNPs involved in two-sample Mendelian rondomization analyses and glucose measurement. | | | |
| --- | --- | --- | --- |
| **Gene** | **OR** | **95%CI** | **P-value** |
| ABCC8+KCNJ11 | 0.934 | [0.844, 1.033] | 0.182 |
| DPP4 | 1.297 | [1.205, 1.396] | **3.559E-12** |
| ETFDH | 0.876 | [0.660, 1.161] | 0.356 |
| GLP1R | 1.657 | [1.443, 1.903] | **9.014E-13** |
| GPD2 | 0.968 | [0.717, 1.306] | 0.831 |
| INSR | 1.040 | [0.819, 1.320] | 0.747 |
| PPARG | 1.033 | [1.002, 1.065] | **0.035** |
| PRKAB1 | 0.894 | [0.798, 1.003] | 0.055 |
| SLC5A2 | 0.937 | [0.891, 0.986] | **0.012** |
| **Abbreviations:** 95% CI: 95% confidence interval; OR: odds ratio; SNP: single nucleotide polymorphisms. | | | |

| **Supplementary Table S6** \| Positive control analysis of SNPs involved in two-sample Mendelian rondomization analyses and type 2 diabetes. | | | |
| --- | --- | --- | --- |
| **Gene** | **OR** | **95%CI** | **P-value** |
| ABCC8+KCNJ11 | 3.561 | [1.526, 8.308] | **3.299E-03** |
| DPP4 | 45.032 | [22.526, 90.025] | **4.623E-27** |
| ETFDH | 11.797 | [3.623, 38.415] | **4.185E-05** |
| GLP1R | 4.316 | [2.752, 6.767] | **1.871E-10** |
| GPD2 | 4.428 | [0.873, 22.474] | 0.073 |
| INSR | 2.183 | [0.428, 11.125] | 0.348 |
| PPARG | 2.229 | [1.847, 2.691] | **6.368E-17** |
| PRKAB1 | 0.989 | [0.550, 1.776] | 0.969 |
| SLC5A2 | 2.825 | [1.932, 4.132] | **8.515E-08** |
| **Abbreviations:** 95% CI: 95% confidence interval; OR: odds ratio; SNP: single nucleotide polymorphisms. | | | |

| **Supplementary Table S7** \| Positive control analysis of eQTLs involved in summary-data-based Mendelian randomization analyses and body mass index. | | | | | | | | |
| --- | --- | --- | --- | --- | --- | --- | --- | --- |
| **Gene** | **Tissue** | **SMR association** | | | | | **HEIDI Test** | |
|  |  | **Beta** | **SE** | **OR** | **95%CI** | **P-Value** | **P-Value** | **Number of SNPs** |
| ABCC8 | Muscle and Skeletal | 0.010 | 0.003 | 1.010 | [1.003, 1.017] | **3.501E-03** | 0.071 | 18 |
| DPP4 | Blood | -0.046 | 0.015 | 0.955 | [0.928, 0.983] | **1.600E-03** | 0.041 | 14 |
| GPD2 | Blood | -0.015 | 0.004 | 0.985 | [0.976, 0.993] | **4.305E-04** | 0.101 | 20 |
| ETFDH | Blood | -0.007 | 0.006 | 0.993 | [0.982, 1.004] | 1.885E-01 | 0.001 | 20 |
| GLP1R | Blood | -0.081 | 0.027 | 0.922 | [0.874, 0.973] | **2.993E-03** | 0.049 | 10 |
| INSR | Blood | 0.059 | 0.025 | 1.061 | [1.010, 1.115] | **1.968E-02** | 0.795 | 10 |
| KCNJ11 | Blood | -0.067 | 0.012 | 0.935 | [0.913, 0.958] | **3.545E-08** | 0.039 | 20 |
| PPARG | Blood | -0.002 | 0.007 | 0.998 | [0.985, 1.012] | 8.140E-01 | 0.052 | 20 |
| PRKAB1 | Blood | 0.009 | 0.004 | 1.009 | [1.001, 1.017] | **3.691E-02** | 0.053 | 16 |
| SLC5A2 | Blood | 0.134 | 0.040 | 1.144 | [1.057, 1.237] | **8.172E-04** | 0.116 | 8 |
| **Abbreviations:** 95% CI: 95% confidence interval; eQTL: expression quantitative trait loci; GWAS: genome-wide association study; HEIDI: heterogeneity in dependent instruments; OR: odds ratio; SE: standard error; SMR: summary-data-based Mendelian rondomization; SNP: single nucleotide polymorphisms. | | | | | | | | |

| **Supplementary Table S8** \| Positive control analysis of eQTLs involved in summary-data-based Mendelian randomization analyses and glucose measurement. | | | | | | | | |
| --- | --- | --- | --- | --- | --- | --- | --- | --- |
| **Gene** | **Tissue** | **SMR association** | | | | | **HEIDI Test** | |
|  |  | **Beta** | **SE** | **OR** | **95%CI** | **P-Value** | **P-Value** | **Number of SNPs** |
| ABCC8 | Muscle and Skeletal | 0.004 | 0.004 | 1.004 | [0.995, 1.012] | 3.858E-01 | 0.001 | 16 |
| DPP4 | Blood | 0.019 | 0.018 | 1.020 | [0.985, 1.055] | 2.705E-01 | 0.009 | 14 |
| GPD2 | Blood | 0.004 | 0.006 | 1.004 | [0.993, 1.015] | 4.920E-01 | 0.607 | 20 |
| ETFDH | Blood | -0.020 | 0.007 | 0.980 | [0.967, 0.993] | **3.027E-03** | 0.677 | 17 |
| GLP1R | Blood | 0.182 | 0.039 | 1.200 | [1.112, 1.295] | **2.847E-06** | 0.000 | 7 |
| INSR | Blood | -0.172 | 0.039 | 0.842 | [0.780, 0.908] | **8.861E-06** | 0.088 | 9 |
| KCNJ11 | Blood | 0.129 | 0.018 | 1.138 | [1.099, 1.179] | **5.826E-13** | 0.012 | 19 |
| PPARG | Blood | 0.024 | 0.009 | 1.025 | [1.006, 1.043] | **7.721E-03** | 0.452 | 20 |
| PRKAB1 | Blood | -0.001 | 0.005 | 0.999 | [0.989, 1.010] | 9.186E-01 | 0.060 | 17 |
| SLC5A2 | Blood | -0.093 | 0.046 | 0.911 | [0.833, 0.997] | **4.210E-02** | 0.402 | 8 |
| **Abbreviations: 95% CI:** 95% confidence interval; eQTL: expression quantitative trait loci; GWAS: genome-wide association study; HEIDI: heterogeneity in dependent instruments; OR: odds ratio; SE: standard error; SMR: summary-data-based Mendelian rondomization; SNP: single nucleotide polymorphisms. | | | | | | | | |

| **Supplementary Table S9** \| Positive control analysis of eQTLs involved in summary-data-based Mendelian randomization analyses and type 2 diabetes. | | | | | | | | |
| --- | --- | --- | --- | --- | --- | --- | --- | --- |
| **Gene** | **Tissue** | **SMR association** | | | | | **HEIDI Test** | |
|  |  | **Beta** | **SE** | **OR** | **95%CI** | **P-Value** | **P-Value** | **Number of SNPs** |
| ABCC8 | Muscle and Skeletal | 0.017 | 0.015 | 1.017 | [0.987, 1.048] | 2.774E-01 | 0.486 | 20 |
| DPP4 | Blood | 0.192 | 0.066 | 1.211 | [1.065, 1.377] | **3.469E-03** | 0.016 | 12 |
| GPD2 | Blood | -0.035 | 0.020 | 0.966 | [0.928, 1.005] | 8.534E-02 | 0.321 | 20 |
| ETFDH | Blood | -0.028 | 0.024 | 0.972 | [0.927, 1.019] | 2.428E-01 | 0.077 | 19 |
| GLP1R | Blood | -0.088 | 0.108 | 0.916 | [0.741, 1.132] | 4.162E-01 | 0.195 | 13 |
| INSR | Blood | -0.383 | 0.128 | 0.682 | [0.531, 0.876] | **2.710E-03** | 0.145 | 17 |
| KCNJ11 | Blood | 0.409 | 0.059 | 1.505 | [1.340, 1.691] | **5.822E-12** | 0.200 | 20 |
| PPARG | Blood | 0.053 | 0.032 | 1.055 | [0.991, 1.123] | 9.604E-02 | 0.024 | 20 |
| PRKAB1 | Blood | 0.010 | 0.018 | 1.010 | [0.975, 1.046] | 5.885E-01 | 0.319 | 13 |
| SLC5A2 | Blood | -0.129 | 0.157 | 0.879 | [0.646, 1.196] | 4.111E-01 | 0.013 | 13 |
| **Abbreviations: 95% CI:** 95% confidence interval; eQTL: expression quantitative trait loci; GWAS: genome-wide association study; HEIDI: heterogeneity in dependent instruments; OR: odds ratio; SE: standard error; SMR: summary-data-based Mendelian rondomization; SNP: single nucleotide polymorphisms. | | | | | | | | |

| **Supplementary Table S10** \| SMR results of glucose-lowering drug target and anal carcinoma. | | | | | | | | |
| --- | --- | --- | --- | --- | --- | --- | --- | --- |
| **Gene** | **eQTL association** | **SMR association** | | | | | **HEIDI Test** | |
|  |  | **Beta** | **SE** | **OR** | **95%CI** | **P-Value** | **P-Value** | **Number of SNPs** |
| ABCC8 | Muscle and Skeletal | 0.566 | 0.272 | 1.762 | [1.034, 3.022] | **0.037** | 0.544 | 20 |
| DPP4 | Blood | -2.304 | 1.118 | 0.100 | [0.011, 0.893] | **0.039** | 0.796 | 20 |
| GDP2 | Blood | 0.286 | 0.355 | 1.331 | [0.663, 2.671] | 0.422 | 0.911 | 20 |
| ETFDH | Blood | -0.677 | 0.419 | 0.508 | [0.223, 1.156] | 0.106 | 0.833 | 20 |
| GLP1R | Blood | -1.705 | 1.940 | 0.182 | [0.004, 8.144] | 0.380 | 0.570 | 17 |
| INSR | Blood | 1.278 | 1.988 | 3.588 | [0.073, 176.740] | 0.520 | 0.486 | 19 |
| KCNJ11 | Blood | -1.535 | 0.916 | 0.215 | [0.036, 1.297] | 0.094 | 0.512 | 20 |
| PPARG | Blood | 0.876 | 0.559 | 2.402 | [0.803, 7.184] | 0.117 | 0.511 | 20 |
| PRKAB1 | Blood | -0.147 | 0.324 | 0.863 | [0.457, 1.630] | 0.650 | 0.431 | 20 |
| SLC5A2 | Blood | 1.940 | 2.711 | 6.957 | [0.034, 1412.287] | 0.474 | 0.428 | 20 |
| **Abbreviations:** 95% CI: 95% confidence interval; eQTL: expression quantitative trait loci; GWAS: genome-wide association study; HEIDI: heterogeneity in dependent instruments; OR: odds ratio; SE: standard error; SMR: summary-data-based Mendelian rondomization; SNP: single nucleotide polymorphisms. | | | | | | | | |

| **Supplementary Table S11** \| SMR results of glucose-lowering drug target and cardia cancer. | | | | | | | | |
| --- | --- | --- | --- | --- | --- | --- | --- | --- |
| **Gene** | **eQTL association** | **SMR association** | | | | | **HEIDI Test** | |
|  |  | **Beta** | **SE** | **OR** | **95%CI** | **P-Value** | **P-Value** | **Number of SNPs** |
| ABCC8 | Muscle and Skeletal | -0.295 | 0.202 | 0.745 | [0.502, 1.106] | 0.144 | 0.335 | 20 |
| DPP4 | Blood | -0.201 | 0.829 | 0.818 | [0.161, 4.150] | 0.808 | 0.681 | 20 |
| GDP2 | Blood | -0.271 | 0.265 | 0.763 | [0.454, 1.282] | 0.306 | 0.990 | 20 |
| ETFDH | Blood | 0.407 | 0.312 | 1.503 | [0.815, 2.773] | 0.192 | 0.271 | 20 |
| GLP1R | Blood | 0.366 | 1.436 | 1.442 | [0.086, 24.060] | 0.799 | 0.375 | 17 |
| INSR | Blood | 1.105 | 1.483 | 3.019 | [0.165, 55.273] | 0.456 | 0.768 | 19 |
| KCNJ11 | Blood | -1.474 | 0.689 | 0.229 | [0.059, 0.884] | **0.032** | 0.665 | 20 |
| PPARG | Blood | 1.034 | 0.418 | 2.813 | [1.239, 6.388] | **0.013** | 0.462 | 20 |
| PRKAB1 | Blood | -0.146 | 0.243 | 0.864 | [0.537, 1.391] | 0.547 | 0.141 | 20 |
| SLC5A2 | Blood | 0.385 | 2.010 | 1.469 | [0.029, 75.494] | 0.848 | 0.768 | 20 |
| **Abbreviations:** 95% CI: 95% confidence interval; eQTL: expression quantitative trait loci; GWAS: genome-wide association study; HEIDI: heterogeneity in dependent instruments; OR: odds ratio; SE: standard error; SMR: summary-data-based Mendelian rondomization; SNP: single nucleotide polymorphisms. | | | | | | | | |

| **Supplementary Table S12** \| SMR results of glucose-lowering drug target and gastric cancer. | | | | | | | | |
| --- | --- | --- | --- | --- | --- | --- | --- | --- |
| **Gene** | **eQTL association** | **SMR association** | | | | | **HEIDI Test** | |
|  |  | **Beta** | **SE** | **OR** | **95%CI** | **P-Value** | **P-Value** | **Number of SNPs** |
| ABCC8 | Muscle and Skeletal | -0.127 | 0.231 | 0.881 | [0.560, 1.385] | 0.583 | 0.440 | 20 |
| DPP4 | Blood | -0.870 | 0.957 | 0.419 | [0.064, 2.735] | 0.363 | 0.863 | 20 |
| GDP2 | Blood | 0.134 | 0.305 | 1.143 | [0.629, 2.078] | 0.661 | 0.891 | 20 |
| ETFDH | Blood | -0.379 | 0.360 | 0.685 | [0.338, 1.387] | 0.293 | 0.176 | 20 |
| GLP1R | Blood | 0.176 | 1.659 | 1.192 | [0.046, 30.772] | 0.916 | 0.996 | 17 |
| INSR | Blood | 0.050 | 1.705 | 1.051 | [0.037, 29.707] | 0.977 | 0.800 | 19 |
| KCNJ11 | Blood | 0.545 | 0.783 | 1.724 | [0.371, 8.004] | 0.487 | 0.910 | 20 |
| PPARG | Blood | 0.015 | 0.479 | 1.015 | [0.397, 2.597] | 0.976 | 0.499 | 20 |
| PRKAB1 | Blood | -0.401 | 0.278 | 0.669 | 0.388, 1.154] | 0.149 | 0.946 | 20 |
| SLC5A2 | Blood | 2.766 | 2.351 | 15.889 | [0.158, 1594.813] | 0.240 | 0.220 | 20 |
| **Abbreviations:** 95% CI: 95% confidence interval; eQTL: expression quantitative trait loci; GWAS: genome-wide association study; HEIDI: heterogeneity in dependent instruments; OR: odds ratio; SE: standard error; SMR: summary-data-based Mendelian rondomization; SNP: single nucleotide polymorphisms. | | | | | | | | |

| **Supplementary Table S13** \| SMR results of glucose-lowering drug target and hepatocellular carcinoma. | | | | | | | | |
| --- | --- | --- | --- | --- | --- | --- | --- | --- |
| **Gene** | **eQTL association** | **SMR association** | | | | | **HEIDI Test** | |
|  |  | **Beta** | **SE** | **OR** | **95%CI** | **P-Value** | **P-Value** | **Number of SNPs** |
| ABCC8 | Muscle and Skeletal | 0.071 | 0.216 | 1.074 | [0.703, 1.640] | 0.742 | 0.610 | 20 |
| DPP4 | Blood | 1.179 | 0.898 | 3.253 | [0.560, 18.904] | 0.189 | 0.168 | 20 |
| GDP2 | Blood | -0.039 | 0.286 | 0.962 | [0.549, 1.685] | 0.892 | 0.784 | 20 |
| ETFDH | Blood | -0.621 | 0.337 | 0.537 | [0.277, 1.040] | 0.065 | 0.945 | 20 |
| GLP1R | Blood | -0.002 | 1.551 | 0.998 | [0.048, 20.864] | 0.999 | 0.893 | 17 |
| INSR | Blood | -0.684 | 1.599 | 0.505 | [0.022, 11.597] | 0.669 | 0.421 | 19 |
| KCNJ11 | Blood | -0.951 | 0.735 | 0.386 | [0.091, 1.631] | 0.196 | 0.686 | 20 |
| PPARG | Blood | -0.091 | 0.450 | 0.913 | [0.378, 2.204] | 0.839 | 0.961 | 20 |
| PRKAB1 | Blood | 0.043 | 0.261 | 1.044 | [0.626, 1.742] | 0.869 | 0.669 | 20 |
| SLC5A2 | Blood | 1.679 | 2.184 | 5.358 | [0.074, 387.050] | 0.442 | 0.129 | 20 |
| **Abbreviations:** 95% CI: 95% confidence interval; eQTL: expression quantitative trait loci; GWAS: genome-wide association study; HEIDI: heterogeneity in dependent instruments; OR: odds ratio; SE: standard error; SMR: summary-data-based Mendelian rondomization; SNP: single nucleotide polymorphisms. | | | | | | | | |

| **Supplementary Table S14** \| SMR results of glucose-lowering drug target and intrahepatic cholangiocarcinoma. | | | | | | | | |
| --- | --- | --- | --- | --- | --- | --- | --- | --- |
| **Gene** | **eQTL association** | **SMR association** | | | | | **HEIDI Test** | |
|  |  | **Beta** | **SE** | **OR** | **95%CI** | **P-Value** | **P-Value** | **Number of SNPs** |
| ABCC8 | Muscle and Skeletal | -0.260 | 0.273 | 0.771 | [0.451, 1.317] | 0.341 | 0.159 | 20 |
| DPP4 | Blood | -0.686 | 1.128 | 0.503 | [0.055, 4.595] | 0.543 | 0.838 | 20 |
| GDP2 | Blood | -0.238 | 0.360 | 0.788 | [0.389, 1.597] | 0.509 | 0.291 | 20 |
| ETFDH | Blood | -0.074 | 0.425 | 0.928 | [0.403, 2.137] | 0.861 | 0.252 | 20 |
| GLP1R | Blood | 3.289 | 1.996 | 26.821 | 0.536, 1342.227] | 0.099 | 0.181 | 17 |
| INSR | Blood | -1.444 | 2.017 | 0.236 | [0.005, 12.299] | 0.474 | 0.169 | 19 |
| KCNJ11 | Blood | 1.045 | 0.926 | 2.844 | [0.463, 17.462] | 0.259 | 0.233 | 20 |
| PPARG | Blood | -0.501 | 0.567 | 0.606 | [0.199, 1.841] | 0.377 | 0.161 | 20 |
| PRKAB1 | Blood | 0.317 | 0.328 | 1.373 | [0.722, 2.611] | 0.334 | 0.669 | 20 |
| SLC5A2 | Blood | -1.793 | 2.743 | 0.167 | [0.001, 35.970] | 0.513 | 0.755 | 20 |
| **Abbreviations:** 95% CI: 95% confidence interval; eQTL: expression quantitative trait loci; GWAS: genome-wide association study; HEIDI: heterogeneity in dependent instruments; OR: odds ratio; SE: standard error; SMR: summary-data-based Mendelian rondomization; SNP: single nucleotide polymorphisms. | | | | | | | | |

| **Supplementary Table S15** \| SMR results of glucose-lowering drug target and rectum cancer. | | | | | | | | |
| --- | --- | --- | --- | --- | --- | --- | --- | --- |
| **Gene** | **eQTL association** | **SMR association** | | | | | **HEIDI Test** | |
|  |  | **Beta** | **SE** | **OR** | **95%CI** | **P-Value** | **P-Value** | **Number of SNPs** |
| ABCC8 | Muscle and Skeletal | 0.082 | 0.073 | 1.086 | [0.941, 1.252] | 0.258 | 0.563 | 20 |
| DPP4 | Blood | -0.119 | 0.300 | 0.888 | [0.493, 1.598] | 0.691 | 0.638 | 20 |
| GDP2 | Blood | 0.090 | 0.096 | 1.094 | [0.907, 1.321] | 0.348 | 0.312 | 20 |
| ETFDH | Blood | 0.103 | 0.113 | 1.109 | [0.888, 1.384] | 0.362 | 0.269 | 20 |
| GLP1R | Blood | 0.108 | 0.520 | 1.114 | [0.402, 3.088] | 0.836 | 0.044 | 17 |
| INSR | Blood | -0.824 | 0.544 | 0.439 | [0.151, 1.273] | 0.130 | 0.189 | 19 |
| KCNJ11 | Blood | 0.179 | 0.246 | 1.196 | [0.738, 1.937] | 0.467 | 0.174 | 20 |
| PPARG | Blood | -0.126 | 0.151 | 0.881 | [0.656, 1.185] | 0.402 | 0.300 | 20 |
| PRKAB1 | Blood | 0.134 | 0.088 | 1.144 | [0.963, 1.358] | 0.125 | 0.219 | 20 |
| SLC5A2 | Blood | 1.283 | 0.757 | 3.608 | [0.818, 15.918] | 0.090 | 0.887 | 20 |
| **Abbreviations:** 95% CI: 95% confidence interval; eQTL: expression quantitative trait loci; GWAS: genome-wide association study; HEIDI: heterogeneity in dependent instruments; OR: odds ratio; SE: standard error; SMR: summary-data-based Mendelian rondomization; SNP: single nucleotide polymorphisms. | | | | | | | | |

| **Supplementary Table S16** \| SMR results of glucose-lowering drug target and pancreatic cancer. | | | | | | | | |
| --- | --- | --- | --- | --- | --- | --- | --- | --- |
| **Gene** | **eQTL association** | **SMR association** | | | | | **HEIDI Test** | |
|  |  | **Beta** | **SE** | **OR** | **95%CI** | **P-Value** | **P-Value** | **Number of SNPs** |
| ABCC8 | Muscle and Skeletal | 0.132 | 0.061 | 1.142 | [1.013, 1.287] | **0.030** | 0.030 | 16 |
| DPP4 | Blood | 0.320 | 0.286 | 1.377 | [0.787, 2.411] | 0.263 | 0.096 | 20 |
| GDP2 | Blood | -0.020 | 0.094 | 0.980 | [0.816, 1.179] | 0.833 | 0.779 | 20 |
| ETFDH | Blood | 0.222 | 0.110 | 1.249 | [1.006, 1.550] | **0.044** | 0.345 | 20 |
| GLP1R | Blood | -0.111 | 0.493 | 0.895 | [0.341, 2.351] | 0.822 | 0.241 | 17 |
| INSR | Blood | 0.144 | 0.454 | 1.155 | [0.474, 2.814] | 0.751 | 0.218 | 19 |
| KCNJ11 | Blood | -0.028 | 0.227 | 0.972 | [0.623, 1.518] | 0.902 | 0.114 | 20 |
| PPARG | Blood | -0.154 | 0.149 | 0.857 | [0.640, 1.148] | 0.301 | 0.189 | 20 |
| PRKAB1 | Blood | -0.225 | 0.095 | 0.798 | [0.662, 0.962] | **0.018** | 0.966 | 20 |
| SLC5A2 | Blood | -0.050 | 0.741 | 0.952 | [0.223, 4.066] | 0.947 | 0.541 | 20 |
| **Abbreviations:** 95% CI: 95% confidence interval; eQTL: expression quantitative trait loci; GWAS: genome-wide association study; HEIDI: heterogeneity in dependent instruments; OR: odds ratio; SE: standard error; SMR: summary-data-based Mendelian rondomization; SNP: single nucleotide polymorphisms. | | | | | | | | |

| **Supplementary Table S17** \| The mediation effects of glucose measurement on glucose-lowering drug target and risks of gastrointestinal cancer. | | | | | |
| --- | --- | --- | --- | --- | --- |
| **Gene** | **Cancer** | **OR** | **95%CI** | **Proportion of mediation effect** | **P-Value** |
| ABCC8+KCNJ11 | Anal Carcinoma | 1.007 | [0.840, 1.208] | 0.089% | 0.937 |
|  | Hepatocellular Carcinoma | 0.963 | [0.450, 2.060] | 0.815% | 0.922 |
|  | Intrahepatic Cholangiocarcinoma | 1.050 | [0.307, 3.589] | 0.804% | 0.938 |
|  | Pancreatic Cancer | 1.025 | [0.850, 1.236] | 1.150% | 0.798 |
| DPP4 | Anal Carcinoma | 0.973 | [0.811, 1.167] | 0.224% | 0.766 |
|  | Intrahepatic Cholangiocarcinoma | 0.831 | [0.243, 2.841] | 2.059% | 0.768 |
| GLP1R | Anal Carcinoma | 0.948 | [0.780, 1.151] | 0.580% | 0.588 |
|  | Cardia Cancer | 0.897 | [0.676, 1.190] | 1.901% | 0.450 |
| GPD2 | Rectum Cancer | 0.994 | [0.911, 1.085] | 0.110% | 0.890 |
| PPARG | Anal Carcinoma | 0.997 | [0.831, 1.194] | 0.093% | 0.970 |
|  | Gastric Cancer | 1.021 | [0.408, 2.555] | 0.745% | 0.965 |
|  | Hepatocellular Carcinoma | 1.018 | [0.476, 2.179] | 0.437% | 0.963 |
|  | Intrahepatic Cholangiocarcinoma | 0.977 | [0.286, 3.339] | 1.062% | 0.970 |
|  | Pancreatic Cancer | 0.988 | [0.819, 1.192] | 0.943% | 0.903 |
| PRKAB1 | Intrahepatic Cholangiocarcinoma | 1.083 | [0.317, 3.700] | 1.259% | 0.899 |
| SLC5A2 | Cardia Cancer | 1.014 | [0.771, 1.334] | 0.374% | 0.920 |
|  | Hepatocellular Carcinoma | 0.964 | [0.451, 2.064] | 0.785% | 0.926 |
|  | Pancreatic Cancer | 1.024 | [0.849, 1.234] | 1.227% | 0.808 |
| **Abbreviations:** 95% CI: 95% confidence interval; OR: odds ratio. | | | | | |

| **Supplementary Table S18** \| The mediation effects of type 2 diabetes on glucose-lowering drug target and risks of gastrointestinal cancer. | | | | | |
| --- | --- | --- | --- | --- | --- |
| **Gene** | **Cancer** | **OR** | **95%CI** | **Proportion of mediation effect** | **P-Value** |
| ABCC8+KCNJ11 | Anal Carcinoma | 1.023 | [0.349, 3.001] | 0.280% | 0.967 |
|  | Hepatocellular Carcinoma | 1.063 | [0.363, 3.119] | 1.313% | 0.911 |
|  | Intrahepatic Cholangiocarcinoma | 0.914 | [0.312, 2.681] | 1.478% | 0.870 |
|  | Pancreatic Cancer | 1.105 | [0.377, 3.241] | 4.685% | 0.856 |
| DPP4 | Anal Carcinoma | 1.071 | [0.077, 14.972] | 0.558% | 0.959 |
|  | Intrahepatic Cholangiocarcinoma | 0.764 | [0.055, 10.675] | 3.001% | 0.841 |
| GLP1R | Anal Carcinoma | 1.027 | [0.532, 1.982] | 0.285% | 0.937 |
|  | Cardia Cancer | 1.004 | [0.520, 1.938] | 0.070% | 0.990 |
| GPD2 | Rectum Cancer | 1.009 | [0.090, 11.312] | 0.157% | 0.994 |
| PPARG | Anal Carcinoma | 1.015 | [0.872, 1.180] | 0.385% | 0.851 |
|  | Gastric Cancer | 0.996 | [0.857, 1.159] | 0.127% | 0.964 |
|  | Hepatocellular Carcinoma | 1.040 | [0.893, 1.210] | 0.934% | 0.616 |
|  | Intrahepatic Cholangiocarcinoma | 0.945 | [0.810, 1.101] | 2.587% | 0.468 |
|  | Pancreatic Cancer | 1.065 | [0.916, 1.239] | 5.093% | 0.413 |
| PRKAB1 | Intrahepatic Cholangiocarcinoma | 1.001 | [0.972, 1.031] | 0.013% | 0.957 |
| SLC5A2 | Cardia Cancer | 1.003 | [0.676, 1.488] | 0.076% | 0.989 |
|  | Hepatocellular Carcinoma | 1.052 | [0.708, 1.561] | 1.091% | 0.803 |
|  | Pancreatic Cancer | 1.085 | [0.731, 1.611] | 4.314% | 0.685 |
| **Abbreviations:** 95% CI: 95% confidence interval; OR: odds ratio. | | | | | |

| **Supplementary Table S19** \| Colocalization analyses of glucose-lowering drug targets and gastrointestinal cancer risks. | | |
| --- | --- | --- |
| **Gastrointestinal Cancer** | **Gene** | **PP4** |
| Anal Carcinoma | DPP4 | **0.856** |
|  | GLP1R | **0.970** |
|  | ABCC8+KCNJ11 | **0.861** |
|  | PPARG | 0.500 |
| Cardia Cancer | GLP1R | 0.500 |
|  | SLC5A2 | 0.223 |
| Gastric Cancer | PPARG | 0.500 |
| Hepatocellular Carcinoma | ABCC8+KCNJ11 | **0.862** |
|  | PPARG | 0.500 |
|  | SLC5A2 | 0.229 |
| Intrahepatic Cholangiocarcinoma | DPP4 | **0.807** |
|  | ABCC8+KCNJ11 | **0.860** |
|  | PPARG | 0.500 |
|  | PRKAB1 | **0.933** |
| Pancreatic Cancer | ABCC8+KCNJ11 | **0.836** |
|  | PPARG | 0.500 |
|  | SLC5A2 | 0.217 |
| Rectum Cancer | GPD2 | **1.000** |

| **Supplementary Table S20** \| Sensitivity and power analyses of two-sample Mendelian randomization analyses in discovery cohorts. | | | | | | | | | | | | | | |
| --- | --- | --- | --- | --- | --- | --- | --- | --- | --- | --- | --- | --- | --- | --- |
| **Gene** | **Cancer** | Number of SNP | MR Egger Method | | | | Weighted Median Method | | | | Heterogeneity Test | Pleiotropy Test | MR-PRESSO Global Test | **Power** |
|  |  |  | Beta | SE | OR | P-Value | Beta | SE | OR | P-Value | P-Value | P-Value | P-Value |  |
| ABCC8+KCNJ11 | Anal Carcinoma | 15 | -9.627 | 7.315 | 6.591E-05 | 0.211 | -7.841 | 3.685 | 3.932E-04 | **0.033** | 0.990 | 0.836 | 0.993 | 0.910 |
|  | Cardia Cancer | 15 | -2.302 | 8.799 | 0.100 | 0.798 | -0.702 | 3.463 | 0.495 | 0.839 | 0.002 | 0.653 | 0.685 | 1.000 |
|  | Gastric Cancer | 15 | 1.341 | 7.368 | 3.823 | 0.858 | -3.094 | 3.636 | 0.045 | 0.395 | 0.197 | 0.608 | 0.212 | 0.960 |
|  | Hepatocellular Carcinoma | 15 | 3.632 | 5.873 | 37.787 | 0.547 | -5.948 | 3.100 | 2.611E-03 | 0.055 | 0.386 | 0.150 | 0.414 | 0.940 |
|  | Intrahepatic Cholangiocarcinoma | 15 | 5.180 | 7.415 | 1.776E+02 | 0.497 | 6.082 | 3.833 | 4.380E+02 | 0.113 | 0.982 | 0.897 | 0.982 | 0.770 |
|  | Pancreatic Cancer | 15 | 1.345 | 2.342 | 3.839 | 0.575 | 2.661 | 1.146 | 14.315 | **2.025E-02** | 0.290 | 0.723 | 0.305 | 1.000 |
|  | Rectum Cancer | 15 | 0.941 | 1.980 | 2.562 | 0.642 | 1.212 | 1.030 | 3.360 | 0.239 | 0.613 | 0.832 | 0.624 | 1.000 |
| DPP4 | Anal Carcinoma | 27 | -36.136 | 7.456 | 2.025E-16 | **5.549E-05** | -11.427 | 3.653 | 1.090E-05 | **1.760E-03** | 0.248 | 0.135 | 0.299 | 1.000 |
|  | Cardia Cancer | 27 | -12.700 | 5.580 | 3.050E-06 | 0.032 | -1.676 | 2.586 | 0.187 | 0.517 | 0.882 | 0.060 | 0.898 | 1.000 |
|  | Gastric Cancer | 27 | -2.273 | 6.359 | 0.103 | 0.724 | -1.923 | 2.990 | 0.146 | 0.520 | 0.853 | 0.918 | 0.850 | 0.920 |
|  | Hepatocellular Carcinoma | 3 | 10.646 | 30.856 | 4.202E+04 | 0.788 | 7.080 | 5.844 | 1.188E+03 | 0.226 | 0.216 | 0.839 | 0.336 | 1.000 |
|  | Intrahepatic Cholangiocarcinoma | 27 | 7.401 | 7.567 | 1.637E+03 | 0.337 | 8.298 | 3.446 | 4.017E+03 | **1.602E-02** | 0.952 | 0.825 | 0.960 | 0.790 |
|  | Pancreatic Cancer | 27 | -7.645 | 2.270 | 4.784E-04 | **2.459E-03** | -1.701 | 1.030 | 0.182 | 0.099 | 0.782 | 0.057 | 0.788 | 1.000 |
|  | Rectum Cancer | 3 | -5.850 | 2.017 | 2.880E-03 | **7.663E-03** | 0.830 | 0.963 | 2.293 | 0.389 | 0.209 | 0.424 | 0.213 | 1.000 |
| ETFDH | Anal Carcinoma | 2 | NA | NA | NA | NA | NA | NA | NA | NA | 0.211 | NA | NA | 0.900 |
|  | Cardia Cancer | 2 | NA | NA | NA | NA | NA | NA | NA | NA | 0.394 | NA | NA | 1.000 |
|  | Gastric Cancer | 2 | NA | NA | NA | NA | NA | NA | NA | NA | 0.458 | NA | NA | 0.670 |
|  | Hepatocellular Carcinoma | 2 | NA | NA | NA | NA | NA | NA | NA | NA | 0.189 | NA | NA | 0.910 |
|  | Intrahepatic Cholangiocarcinoma | 2 | NA | NA | NA | NA | NA | NA | NA | NA | 0.380 | NA | NA | 0.850 |
|  | Pancreatic Cancer | 2 | NA | NA | NA | NA | NA | NA | NA | NA | 0.677 | NA | NA | 1.000 |
|  | Rectum Cancer | 2 | NA | NA | NA | NA | NA | NA | NA | NA | 0.782 | NA | NA | 1.000 |
| GLP1R | Anal Carcinoma | 23 | 2.591 | 8.862 | 13.338 | 0.773 | 8.463 | 4.407 | 4.737E+03 | 0.055 | 0.443 | 0.264 | 0.494 | 0.730 |
|  | Cardia Cancer | 23 | 5.804 | 5.696 | 3.315E+02 | 0.320 | 4.671 | 3.203 | 1.068E+02 | 0.145 | 0.241 | 0.990 | 0.275 | 0.670 |
|  | Gastric Cancer | 23 | 8.583 | 5.844 | 5.338E+03 | 0.157 | 4.665 | 3.354 | 1.061E+02 | 0.164 | 0.877 | 0.306 | 0.909 | 1.000 |
|  | Hepatocellular Carcinoma | 23 | -1.401 | 5.486 | 0.246 | 0.801 | -2.105 | 3.013 | 0.122 | 0.485 | 0.993 | 0.742 | 0.995 | 0.920 |
|  | Intrahepatic Cholangiocarcinoma | 23 | 1.262 | 6.883 | 3.534 | 0.856 | 4.314 | 3.694 | 7.476E+01 | 0.243 | 0.968 | 0.652 | 0.978 | 1.000 |
|  | Pancreatic Cancer | 23 | -0.258 | 1.761 | 7.725E-01 | 0.885 | 0.670 | 0.974 | 1.954 | 0.491 | 0.979 | 0.835 | 0.984 | 0.940 |
|  | Rectum Cancer | 23 | 1.109 | 2.169 | 3.030 | 0.615 | 1.015 | 1.155 | 2.761 | 0.379 | 0.140 | 0.862 | 0.161 | 1.000 |
| GPD2 | Anal Carcinoma | 2 | NA | NA | NA | NA | NA | NA | NA | NA | 0.123 | NA | NA | 0.670 |
|  | Cardia Cancer | 2 | NA | NA | NA | NA | NA | NA | NA | NA | 0.910 | NA | NA | 0.960 |
|  | Gastric Cancer | 2 | NA | NA | NA | NA | NA | NA | NA | NA | 0.852 | NA | NA | 0.520 |
|  | Hepatocellular Carcinoma | 2 | NA | NA | NA | NA | NA | NA | NA | NA | 0.939 | NA | NA | 0.980 |
|  | Intrahepatic Cholangiocarcinoma | 2 | NA | NA | NA | NA | NA | NA | NA | NA | 0.776 | NA | NA | 0.510 |
|  | Pancreatic Cancer | 2 | NA | NA | NA | NA | NA | NA | NA | NA | 0.740 | NA | NA | 1.000 |
|  | Rectum Cancer | 2 | NA | NA | NA | NA | NA | NA | NA | NA | 0.417 | NA | NA | 1.000 |
| INSR | Anal Carcinoma | 4 | 42.969 | 73.848 | 4.581E+18 | 0.620 | 9.289 | 8.329 | 1.082E+04 | 0.265 | 0.766 | 0.673 | 0.764 | 0.530 |
|  | Cardia Cancer | 4 | -23.687 | 71.988 | 5.162E-11 | 0.773 | 7.724 | 6.752 | 2.262E+03 | 0.253 | 0.292 | 0.697 | 0.349 | 0.560 |
|  | Gastric Cancer | 4 | 16.955 | 85.213 | 2.310E+07 | 0.861 | 2.199 | 7.723 | 9.019 | 0.776 | 0.296 | 0.832 | 0.333 | 0.980 |
|  | Hepatocellular Carcinoma | 4 | -29.205 | 59.627 | 2.073E-13 | 0.673 | -3.591 | 6.641 | 2.756E-02 | 0.589 | 0.887 | 0.702 | 0.889 | 0.920 |
|  | Intrahepatic Cholangiocarcinoma | 4 | -22.692 | 75.089 | 1.397E-10 | 0.791 | -7.065 | 8.570 | 8.548E-04 | 0.410 | 0.985 | 0.852 | 0.986 | 0.900 |
|  | Pancreatic Cancer | 5 | -2.470 | 26.229 | 8.460E-02 | 0.931 | -0.177 | 2.622 | 0.837 | 0.946 | 0.296 | 0.957 | 0.311 | 0.720 |
|  | Rectum Cancer | 4 | -10.201 | 20.049 | 3.715E-05 | 0.661 | -0.509 | 2.311 | 0.601 | 0.826 | 0.865 | 0.672 | 0.864 | 1.000 |
| PPARG | Anal Carcinoma | 58 | 1.185 | 3.193 | 3.270 | 0.712 | 3.380 | 1.463 | 29.379 | **0.021** | 0.744 | 0.400 | 0.759 | 1.000 |
|  | Cardia Cancer | 58 | -1.797 | 2.628 | 1.658E-01 | 0.497 | 0.884 | 1.091 | 2.421 | 0.418 | 0.132 | 0.390 | 0.173 | 0.920 |
|  | Gastric Cancer | 58 | -4.844 | 2.742 | 7.879E-03 | 0.083 | -1.046 | 1.250 | 3.513E-01 | 0.403 | 0.800 | 0.427 | 0.810 | 0.970 |
|  | Hepatocellular Carcinoma | 58 | 2.877 | 2.571 | 1.776E+01 | 0.268 | 3.750 | 1.105 | 4.251E+01 | **6.929E-04** | 0.999 | 0.603 | 1.000 | 0.870 |
|  | Intrahepatic Cholangiocarcinoma | 58 | 3.497 | 3.249 | 3.302E+01 | 0.286 | 3.715 | 1.467 | 4.107E+01 | **0.011** | 0.490 | 0.675 | 0.505 | 1.000 |
|  | Pancreatic Cancer | 63 | -0.536 | 0.868 | 5.851E-01 | 0.539 | -1.116 | 0.410 | 3.276E-01 | **6.538E-03** | 0.502 | 0.397 | 0.527 | 0.830 |
|  | Rectum Cancer | 9 | 4.554 | 2.602 | 9.498E+01 | 0.124 | 1.279 | 0.688 | 3.593 | 0.063 | 0.232 | 0.174 | 0.224 | 1.000 |
| PRKAB1 | Anal Carcinoma | 21 | -4.264 | 8.665 | 1.406E-02 | 0.628 | -3.207 | 2.967 | 4.047E-02 | 0.280 | 0.934 | 0.914 | 0.945 | 0.990 |
|  | Cardia Cancer | 21 | -6.939 | 6.470 | 9.694E-04 | 0.297 | 2.153 | 2.186 | 8.609 | 0.325 | 0.799 | 0.128 | 0.833 | 1.000 |
|  | Gastric Cancer | 21 | 4.854 | 7.447 | 1.283E+02 | 0.522 | 1.866 | 2.490 | 6.460 | 0.454 | 0.752 | 0.407 | 0.778 | 0.850 |
|  | Hepatocellular Carcinoma | 21 | -3.317 | 6.968 | 3.626E-02 | 0.639 | -0.912 | 2.385 | 4.018E-01 | 0.702 | 0.700 | 0.886 | 0.729 | 0.890 |
|  | Intrahepatic Cholangiocarcinoma | 21 | 3.777 | 9.002 | 4.368E+01 | 0.679 | 6.030 | 3.105 | 4.158E+02 | 0.052 | 0.458 | 0.775 | 0.506 | 0.880 |
|  | Pancreatic Cancer | 22 | 4.186 | 3.167 | 6.576E+01 | 0.201 | -0.851 | 0.984 | 4.271E-01 | 0.387 | 0.064 | 0.143 | 0.761 | 1.000 |
|  | Rectum Cancer | 21 | 2.717 | 2.341 | 1.514E+01 | 0.260 | 0.315 | 0.761 | 1.371 | 0.679 | 0.971 | 0.427 | 0.978 | 1.000 |
| SLC5A2 | Anal Carcinoma | 23 | 3.007 | 6.103 | 2.023E+01 | 0.627 | -0.654 | 2.545 | 5.197E-01 | 0.797 | 0.775 | 0.410 | 0.797 | 0.970 |
|  | Cardia Cancer | 23 | 3.040 | 4.560 | 2.091E+01 | 0.512 | 3.598 | 1.930 | 3.652E+01 | 0.062 | 0.879 | 0.868 | 0.904 | 1.000 |
|  | Gastric Cancer | 23 | 10.620 | 5.768 | 4.096E+04 | 0.080 | 2.223 | 2.331 | 9.232 | 0.340 | 0.113 | 0.823 | 0.136 | 0.840 |
|  | Hepatocellular Carcinoma | 23 | 5.306 | 6.007 | 2.016E+02 | 0.387 | 6.722 | 2.193 | 8.308E+02 | **2.178E-03** | 0.090 | 0.904 | 0.108 | 0.760 |
|  | Intrahepatic Cholangiocarcinoma | 23 | -8.835 | 7.361 | 1.455E-04 | 0.243 | 5.037 | 2.715 | 1.540E+02 | 0.064 | 0.099 | 0.085 | 0.565 | 1.000 |
|  | Pancreatic Cancer | 25 | -0.735 | 1.667 | 4.797E-01 | 0.664 | 1.673 | 0.709 | 5.330 | **1.828E-02** | 0.859 | 0.112 | 0.869 | 1.000 |
|  | Rectum Cancer | 23 | -1.881 | 1.783 | 1.525E-01 | 0.303 | 0.434 | 0.745 | 1.544 | 0.560 | 0.237 | 0.223 | 0.280 | 0.920 |
| **Abbreviations:** SE: standard error; OR: odds ratio. | | | | | | | | | | | | | |  |

| **Supplementary Table S21** \| Sensitivity and power analyses of two-sample Mendelian randomization analyses in validation cohorts. | | | | | | | | | | | | | | |
| --- | --- | --- | --- | --- | --- | --- | --- | --- | --- | --- | --- | --- | --- | --- |
| **Gene** | **Cancer** | Number of SNP | MR Egger Method | | | | Weighted Median Method | | | | Heterogeneity Test | Pleiotropy Test | MR-PRESSO Global Test | **Power** |
|  |  |  | Beta | SE | OR | P-Value | Beta | SE | OR | P-Value | P-Value | P-Value | P-Value |  |
| ABCC8+KCNJ11 | Anal Carcinoma | 14 | 3.677 | 13.348 | 39.525 | 0.788 | -17.500 | 5.615 | 2.511E-08 | **0.002** | 0.130 | 0.219 | 0.251 | 0.880 |
|  | Cardia Cancer | 15 | -2.869 | 2.403 | 0.057 | 0.254 | -1.292 | 1.247 | 0.275 | 0.300 | 0.437 | 0.340 | 0.450 | 1.000 |
|  | Gastric Cancer | 15 | -3.234 | 1.383 | 0.039 | **0.036** | -0.928 | 0.623 | 0.395 | 0.136 | 0.306 | 0.478 | 0.220 | 0.930 |
|  | Hepatocellular Carcinoma | 14 | -5.498 | 3.854 | 0.004 | 0.179 | -6.949 | 1.949 | 9.597E-04 | **3.631E-04** | 0.509 | 0.783 | 0.570 | 1.000 |
|  | Intrahepatic Cholangiocarcinoma | 14 | 0.628 | 2.760 | 1.874 | 0.824 | 4.351 | 1.442 | 77.519 | **0.003** | 0.202 | 0.207 | 0.250 | 0.870 |
|  | Pancreatic Cancer | 15 | -1.232 | 3.133 | 0.292 | 0.700 | 4.360 | 1.717 | 78.228 | **0.011** | 0.319 | 0.092 | 0.340 | 0.860 |
|  | Rectum Cancer | 14 | -2.454 | 1.851 | 0.086 | 0.210 | -2.351 | 0.984 | 0.095 | **0.017** | 0.366 | 0.504 | 0.310 | 1.000 |
| DPP4 | Anal Carcinoma | 27 | -12.655 | 8.504 | 3.190E-06 | 0.149 | -7.892 | 3.852 | 3.736E-04 | **0.040** | 0.509 | 0.180 | 0.536 | 0.850 |
|  | Cardia Cancer | 26 | -7.913 | 2.711 | 3.660E-04 | **0.008** | -4.357 | 1.198 | 0.013 | **2.751E-04** | 0.173 | 0.063 | 0.570 | 1.000 |
|  | Gastric Cancer | 27 | -0.861 | 2.347 | 0.423 | 0.717 | 0.480 | 0.719 | 1.617 | 0.504 | 0.149 | 0.638 | 0.140 | 1.000 |
|  | Hepatocellular Carcinoma | 27 | 0.783 | 3.726 | 2.188 | 0.835 | 4.002 | 1.616 | 54.686 | 0.133 | 0.987 | 0.440 | 0.990 | 1.000 |
|  | Intrahepatic Cholangiocarcinoma | 27 | -6.148 | 2.435 | 2.137E-03 | **0.018** | 0.542 | 1.092 | 1.720 | 0.620 | 0.491 | 0.311 | 0.480 | 1.000 |
|  | Pancreatic Cancer | 27 | -6.604 | 3.697 | 1.354E-03 | 0.086 | -1.652 | 1.632 | 0.192 | 0.311 | 0.104 | 0.255 | 0.060 | 0.990 |
|  | Rectum Cancer | 27 | 6.239 | 1.700 | 512.191 | 0.115 | 1.800 | 0.784 | 6.050 | 0.216 | 0.636 | 0.166 | 0.63 | 1.000 |
| ETFDH | Anal Carcinoma | 2 | NA | NA | NA | NA | NA | NA | NA | NA | 0.104 | NA | NA | 0.680 |
|  | Cardia Cancer | 2 | NA | NA | NA | NA | NA | NA | NA | NA | 0.293 | NA | NA | 0.920 |
|  | Gastric Cancer | 2 | NA | NA | NA | NA | NA | NA | NA | NA | 0.821 | NA | NA | 1.000 |
|  | Hepatocellular Carcinoma | 2 | NA | NA | NA | NA | NA | NA | NA | NA | 0.484 | NA | NA | 1.000 |
|  | Intrahepatic Cholangiocarcinoma | 2 | NA | NA | NA | NA | NA | NA | NA | NA | 0.296 | NA | NA | 1.000 |
|  | Pancreatic Cancer | 2 | NA | NA | NA | NA | NA | NA | NA | NA | 0.744 | NA | NA | 1.000 |
|  | Rectum Cancer | 2 | NA | NA | NA | NA | NA | NA | NA | NA | 0.832 | NA | NA | 0.670 |
| GLP1R | Anal Carcinoma | 20 | 0.482 | 7.934 | 1.619 | 0.952 | -1.437 | 4.344 | 0.238 | 0.741 | 0.277 | 0.582 | 0.296 | 1.000 |
|  | Cardia Cancer | 23 | 5.057 | 2.131 | 157.190 | **0.027** | 0.959 | 1.240 | 2.610 | 0.439 | 0.742 | 0.302 | 0.760 | 1.000 |
|  | Gastric Cancer | 23 | -0.342 | 0.848 | 0.710 | 0.691 | -0.409 | 0.455 | 0.664 | 0.368 | 0.809 | 0.753 | 0.860 | 1.000 |
|  | Hepatocellular Carcinoma | 20 | -3.546 | 3.223 | 0.029 | 0.286 | 0.479 | 1.800 | 1.615 | 0.790 | 0.900 | 0.142 | 0.880 | 1.000 |
|  | Intrahepatic Cholangiocarcinoma | 20 | -3.048 | 2.068 | 0.047 | 0.158 | 0.279 | 1.214 | 1.321 | 0.818 | 0.450 | 0.127 | 0.410 | 0.530 |
|  | Pancreatic Cancer | 23 | 5.413 | 2.908 | 224.412 | 0.077 | 1.561 | 1.701 | 4.765 | 0.359 | 0.533 | 0.213 | 0.620 | 1.000 |
|  | Rectum Cancer | 23 | 1.980 | 1.644 | 7.243 | 0.244 | 0.411 | 0.823 | 1.509 | 0.617 | 0.163 | 0.233 | 0.170 | 1.000 |
| GPD2 | Anal Carcinoma | 2 | NA | NA | NA | NA | NA | NA | NA | NA | 0.774 | NA | NA | 1.000 |
|  | Cardia Cancer | 2 | NA | NA | NA | NA | NA | NA | NA | NA | 0.693 | NA | NA | 1.000 |
|  | Gastric Cancer | 2 | NA | NA | NA | NA | NA | NA | NA | NA | 0.697 | NA | NA | 1.000 |
|  | Hepatocellular Carcinoma | 2 | NA | NA | NA | NA | NA | NA | NA | NA | 0.545 | NA | NA | 1.000 |
|  | Intrahepatic Cholangiocarcinoma | 2 | NA | NA | NA | NA | NA | NA | NA | NA | 0.169 | NA | NA | 1.000 |
|  | Pancreatic Cancer | 2 | NA | NA | NA | NA | NA | NA | NA | NA | 0.287 | NA | NA | 1.000 |
|  | Rectum Cancer | 2 | NA | NA | NA | NA | NA | NA | NA | NA | 0.797 | NA | NA | 1.000 |
| INSR | Anal Carcinoma | 5 | -7.419 | 74.170 | 5.997E-04 | 0.927 | -4.273 | 8.219 | 0.014 | 0.603 | 0.866 | 0.936 | 0.851 | 0.780 |
|  | Cardia Cancer | 4 | -5.499 | 23.107 | 0.004 | 0.834 | 4.043 | 2.717 | 56.976 | 0.137 | 0.923 | 0.704 | 0.940 | 1.000 |
|  | Gastric Cancer | 5 | -19.382 | 13.469 | 3.825E-09 | 0.246 | 0.931 | 1.710 | 2.537 | 0.586 | 0.538 | 0.231 | 0.550 | 1.000 |
|  | Hepatocellular Carcinoma | 5 | 28.422 | 32.573 | 2.206E+12 | 0.447 | -2.740 | 3.818 | 0.065 | 0.473 | 0.677 | 0.408 | 0.700 | 1.000 |
|  | Intrahepatic Cholangiocarcinoma | 5 | 17.306 | 21.082 | 3.279E+07 | 0.472 | -2.996 | 2.590 | 0.050 | 0.247 | 0.459 | 0.398 | 0.470 | 1.000 |
|  | Pancreatic Cancer | 4 | 27.195 | 31.721 | 6.464E+11 | 0.482 | 2.505 | 3.682 | 12.249 | 0.496 | 0.635 | 0.514 | 0.570 | 1.000 |
|  | Rectum Cancer | 5 | -11.293 | 14.666 | 1.246E-05 | 0.497 | 0.019 | 1.650 | 1.020 | 0.991 | 0.732 | 0.526 | 0.740 | 1.000 |
| PPARG | Anal Carcinoma | 61 | 5.529 | 3.048 | 251.772 | 0.075 | 0.759 | 1.451 | 2.136 | 0.601 | 0.891 | 0.149 | 0.860 | 1.000 |
|  | Cardia Cancer | 61 | 1.866 | 1.004 | 6.462 | 0.068 | 2.356 | 0.462 | 10.553 | 0.341 | 0.682 | 0.578 | 0.740 | 1.000 |
|  | Gastric Cancer | 63 | -1.125 | 0.607 | 0.325 | 0.069 | -0.025 | 0.239 | 0.975 | 0.917 | 0.182 | 0.151 | 0.220 | 1.000 |
|  | Hepatocellular Carcinoma | 61 | -2.425 | 1.365 | 0.088 | 0.081 | -1.010 | 0.661 | 0.364 | 0.126 | 0.936 | 0.276 | 0.920 | 1.000 |
|  | Intrahepatic Cholangiocarcinoma | 61 | 2.284 | 0.881 | 9.819 | **0.012** | 0.316 | 0.428 | 1.372 | 0.061 | 0.704 | 0.388 | 0.750 | 1.000 |
|  | Pancreatic Cancer | 58 | -5.327 | 1.365 | 0.005 | **2.575E-04** | -4.400 | 0.601 | 0.012 | **2.516E-13** | 0.978 | 0.582 | 0.990 | 1.000 |
|  | Rectum Cancer | 61 | -0.468 | 0.629 | 0.626 | 0.460 | 0.555 | 0.305 | 1.741 | 0.069 | 0.352 | 0.156 | 0.330 | 1.000 |
| PRKAB1 | Anal Carcinoma | 22 | -7.216 | 9.369 | 7.345E-04 | 0.450 | -8.258 | 3.158 | 2.593E-04 | 0.089 | 0.522 | 0.124 | 0.500 | 0.690 |
|  | Cardia Cancer | 20 | 0.226 | 2.715 | 1.254 | 0.935 | 1.482 | 0.965 | 4.401 | 0.125 | 0.886 | 0.724 | 0.840 | 1.000 |
|  | Gastric Cancer | 22 | -3.896 | 1.457 | 0.020 | **0.015** | -0.218 | 0.421 | 0.804 | 0.604 | 0.295 | 0.122 | 0.310 | 0.640 |
|  | Hepatocellular Carcinoma | 22 | -7.152 | 4.953 | 7.835E-04 | 0.164 | 0.767 | 1.433 | 2.154 | 0.592 | 0.105 | 0.077 | 0.060 | 1.000 |
|  | Intrahepatic Cholangiocarcinoma | 22 | 1.626 | 2.794 | 5.082 | 0.567 | 1.466 | 0.881 | 4.334 | 0.096 | 0.408 | 0.626 | 0.400 | 1.000 |
|  | Pancreatic Cancer | 21 | 0.007 | 3.702 | 1.007 | 0.998 | -1.644 | 1.227 | 0.193 | 0.180 | 0.892 | 0.545 | 0.900 | 1.000 |
|  | Rectum Cancer | 22 | -0.578 | 2.524 | 0.561 | 0.821 | 0.738 | 0.679 | 2.092 | 0.277 | 0.184 | 0.462 | 0.330 | 1.000 |
| SLC5A2 | Anal Carcinoma | 23 | -0.679 | 6.449 | 0.507 | 0.917 | 0.831 | 2.745 | 2.295 | 0.762 | 0.494 | 0.960 | 0.600 | 0.980 |
|  | Cardia Cancer | 23 | 3.179 | 1.983 | 24.011 | 0.124 | 1.964 | 0.801 | 1.483 | **0.014** | 0.748 | 0.685 | 0.760 | 1.000 |
|  | Gastric Cancer | 25 | 2.461 | 1.093 | 11.719 | 0.341 | 1.514 | 0.402 | 4.545 | 0.166 | 0.081 | 0.201 | 0.820 | 0.430 |
|  | Hepatocellular Carcinoma | 23 | 3.385 | 1.859 | 29.530 | 0.250 | 0.060 | 1.204 | 1.062 | 0.096 | 0.797 | 0.228 | 0.810 | 1.000 |
|  | Intrahepatic Cholangiocarcinoma | 23 | -1.797 | 1.846 | 0.166 | 0.342 | 0.314 | 0.749 | 1.369 | 0.675 | 0.962 | 0.282 | 0.970 | 0.880 |
|  | Pancreatic Cancer | 23 | -1.253 | 2.815 | 0.286 | 0.661 | 3.196 | 1.189 | 24.434 | **0.007** | 0.208 | 0.152 | 0.270 | 1.000 |
|  | Rectum Cancer | 23 | -1.496 | 1.291 | 0.224 | 0.259 | -0.117 | 0.525 | 0.890 | 0.824 | 0.834 | 0.209 | 0.870 | 1.000 |
| **Abbreviations:** SE: standard error; OR: odds ratio. | | | | | | | | | | | | | |  |

**Supplementary Figure S1 |** Regional Manhattan plot of associations of DPP4 and risk of anal carcinoma. The lead SNP is shown as a purple diamond. SNPs within ±500 kb of the glucose-lowering drug target quantitative trait locus were included; p12=1×10^−5^, prior probability a SNP is associated with both DPP4 and anal carcinoma.

**Supplementary Figure S2 |** Regional Manhattan plot of associations of GLP1R and risk of anal carcinoma. The lead SNP is shown as a purple diamond. SNPs within ±500 kb of the glucose-lowering drug target quantitative trait locus were included; p12=1×10^−5^, prior probability a SNP is associated with both GLP1R and anal carcinoma.

**Supplementary Figure S3 |** Regional Manhattan plot of associations of ABCC8+KCNJ11 and risk of anal carcinoma. The lead SNP is shown as a purple diamond. SNPs within ±500 kb of the glucose-lowering drug target quantitative trait locus were included; p12=1×10^−5^, prior probability a SNP is associated with both ABCC8+KCNJ11 and anal carcinoma.

**Supplementary Figure S4 |** Regional Manhattan plot of associations of ABCC8+KCNJ11 and risk of hepatocellular carcinoma. The lead SNP is shown as a purple diamond. SNPs within ±500 kb of the glucose-lowering drug target quantitative trait locus were included; p12=1×10^−5^, prior probability a SNP is associated with both ABCC8+KCNJ11 and hepatocellular carcinoma.

**Supplementary Figure S5 |** Regional Manhattan plot of associations of DPP4 and risk of intrahepatic cholangiocarcinoma. The lead SNP is shown as a purple diamond. SNPs within ±500 kb of the glucose-lowering drug target quantitative trait locus were included; p12=1×10^−5^, prior probability a SNP is associated with both DPP4 and intrahepatic cholangiocarcinoma.

**Supplementary Figure S6 |** Regional Manhattan plot of associations of ABCC8+KCNJ11 and risk of intrahepatic cholangiocarcinoma. The lead SNP is shown as a purple diamond. SNPs within ±500 kb of the glucose-lowering drug target quantitative trait locus were included; p12=1×10^−5^, prior probability a SNP is associated with both ABCC8+KCNJ11 and intrahepatic cholangiocarcinoma.

**Supplementary Figure S7 |** Regional Manhattan plot of associations of PRKAB1 and risk of intrahepatic cholangiocarcinoma. The lead SNP is shown as a purple diamond. SNPs within ±500 kb of the glucose-lowering drug target quantitative trait locus were included; p12=1×10^−5^, prior probability a SNP is associated with both PRKAB1 and intrahepatic cholangiocarcinoma.

**Supplementary Figure S8 |** Regional Manhattan plot of associations of GPD2 and risk of rectum cancer. The lead SNP is shown as a purple diamond. SNPs within ±500 kb of the glucose-lowering drug target quantitative trait locus were included; p12=1×10^−5^, prior probability a SNP is associated with both GPD2 and rectum cancer.


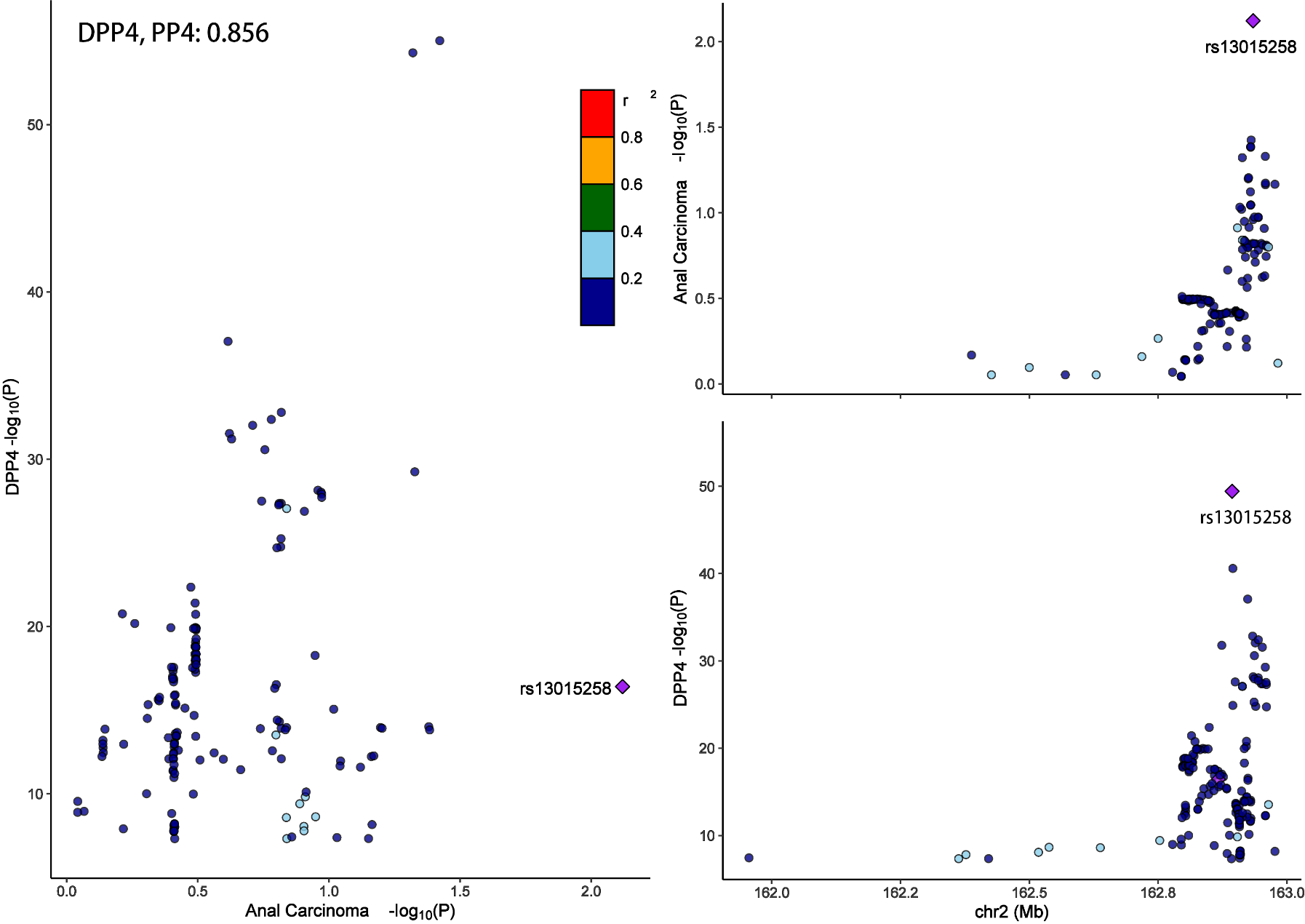

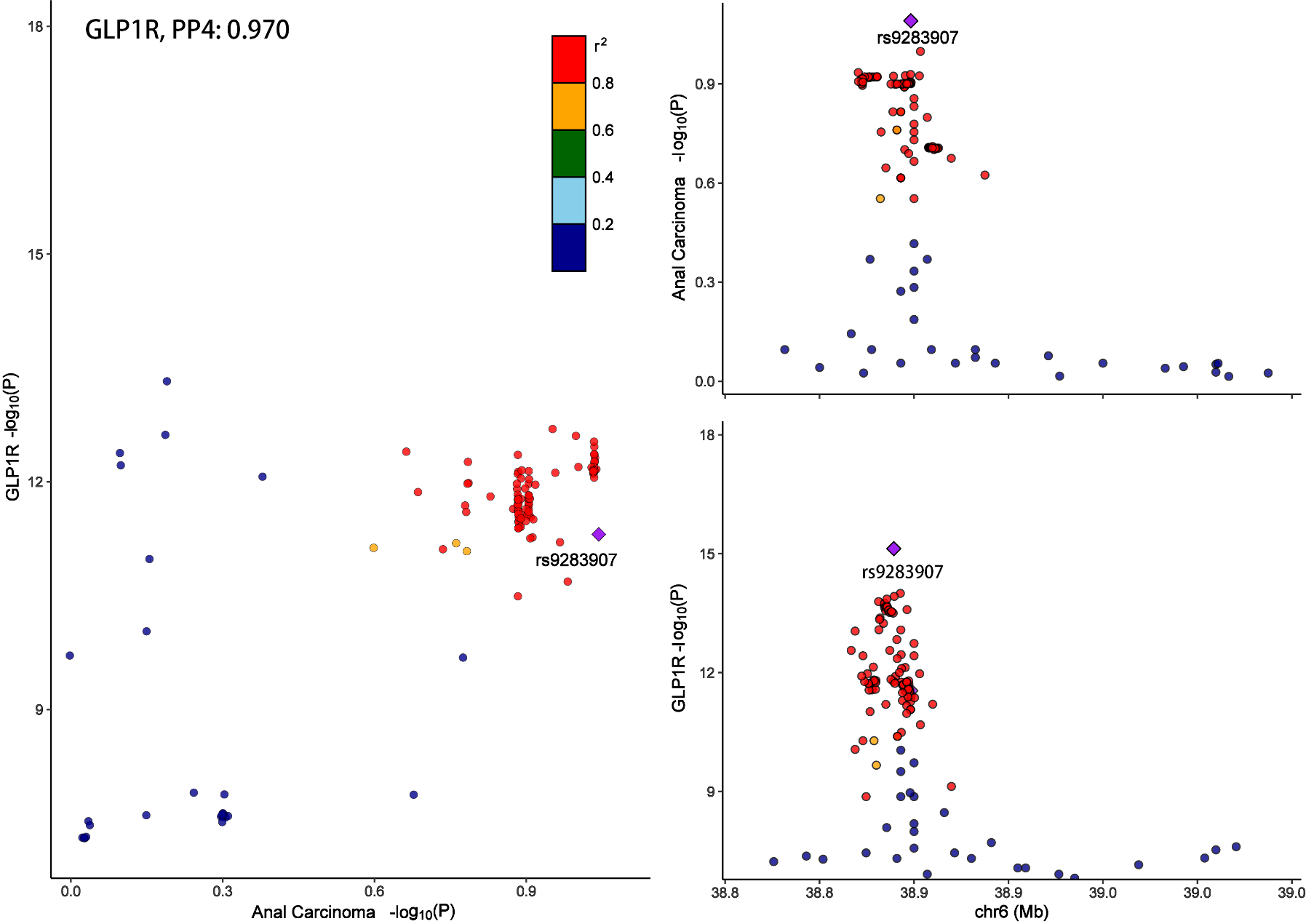

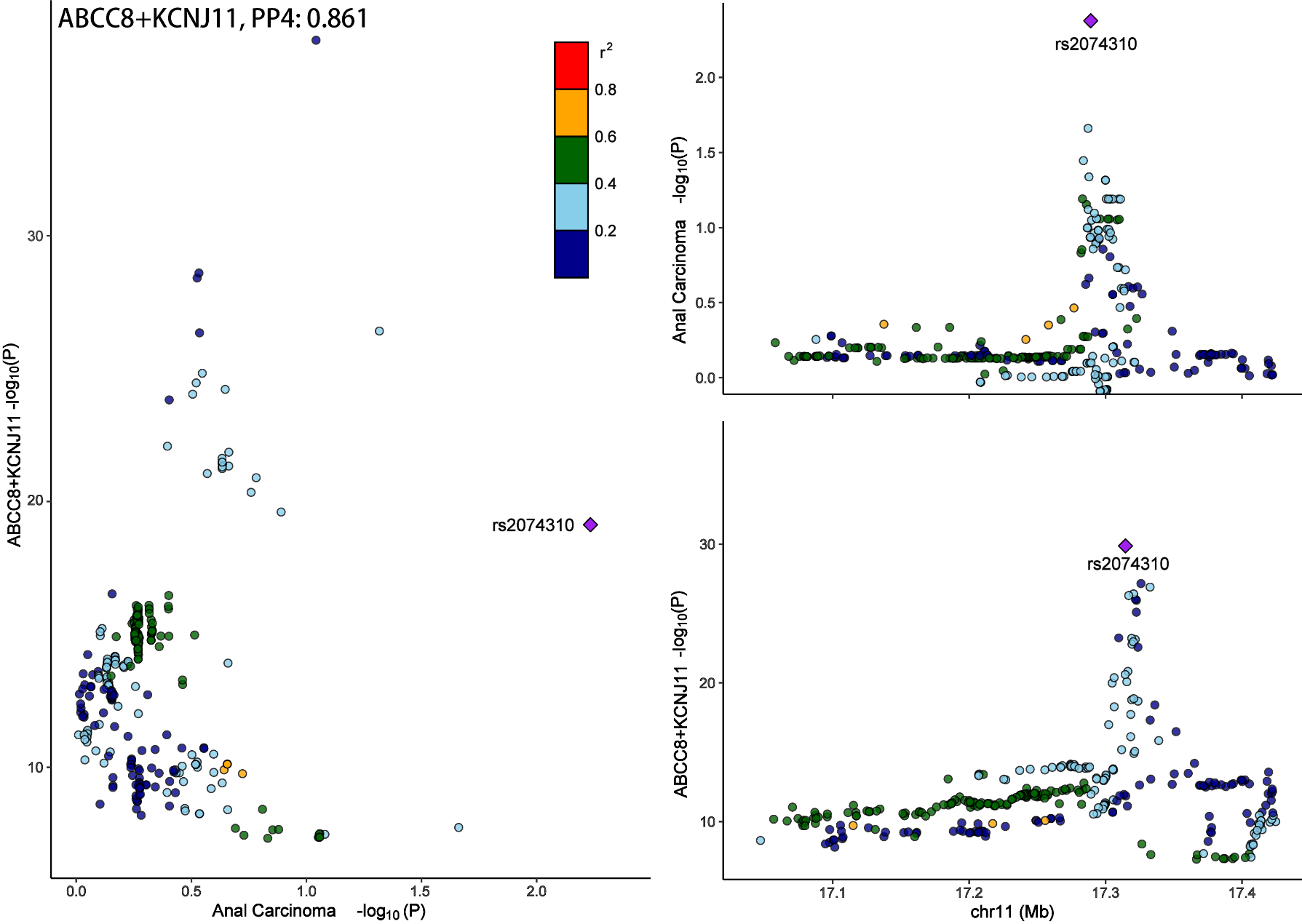

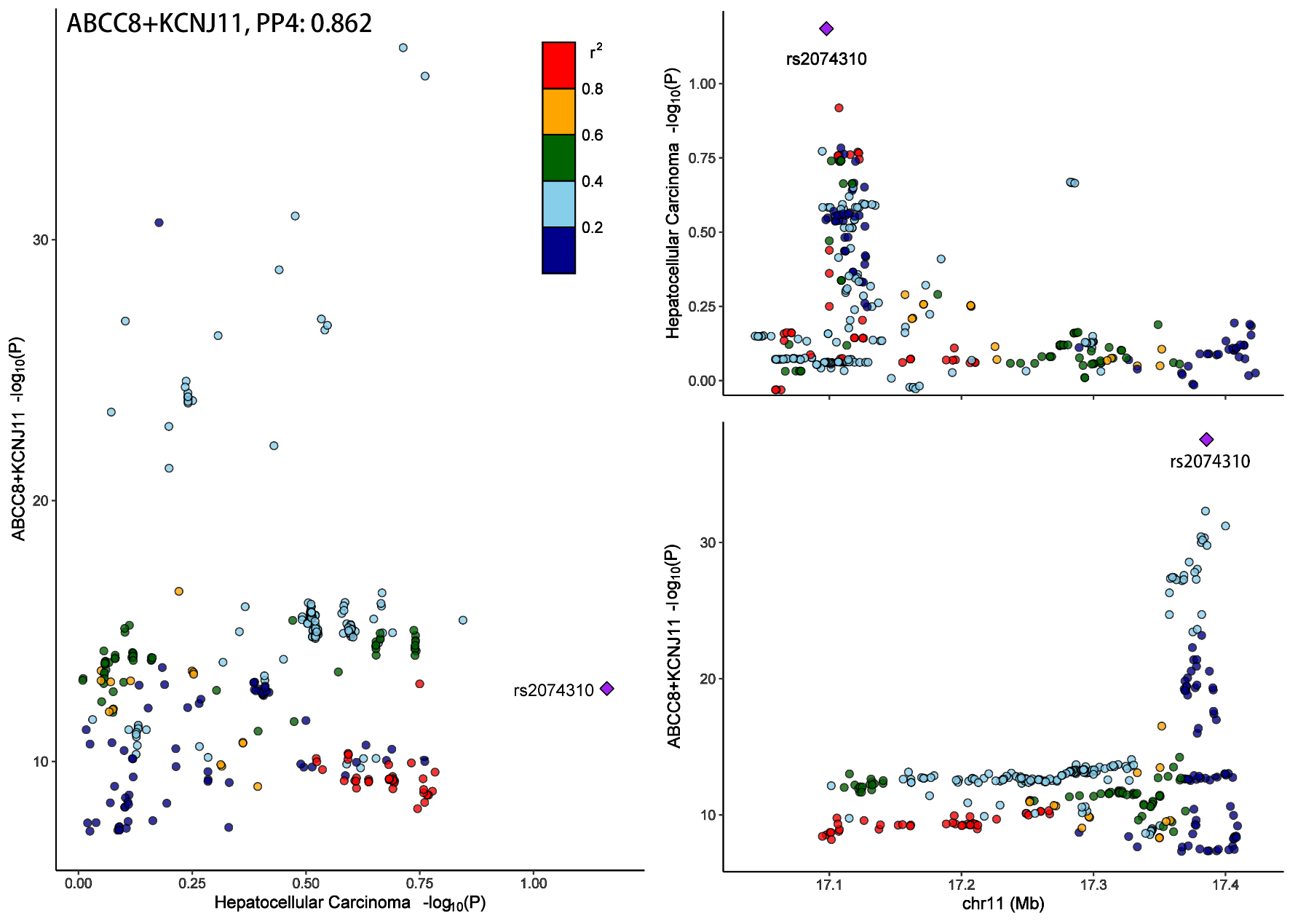

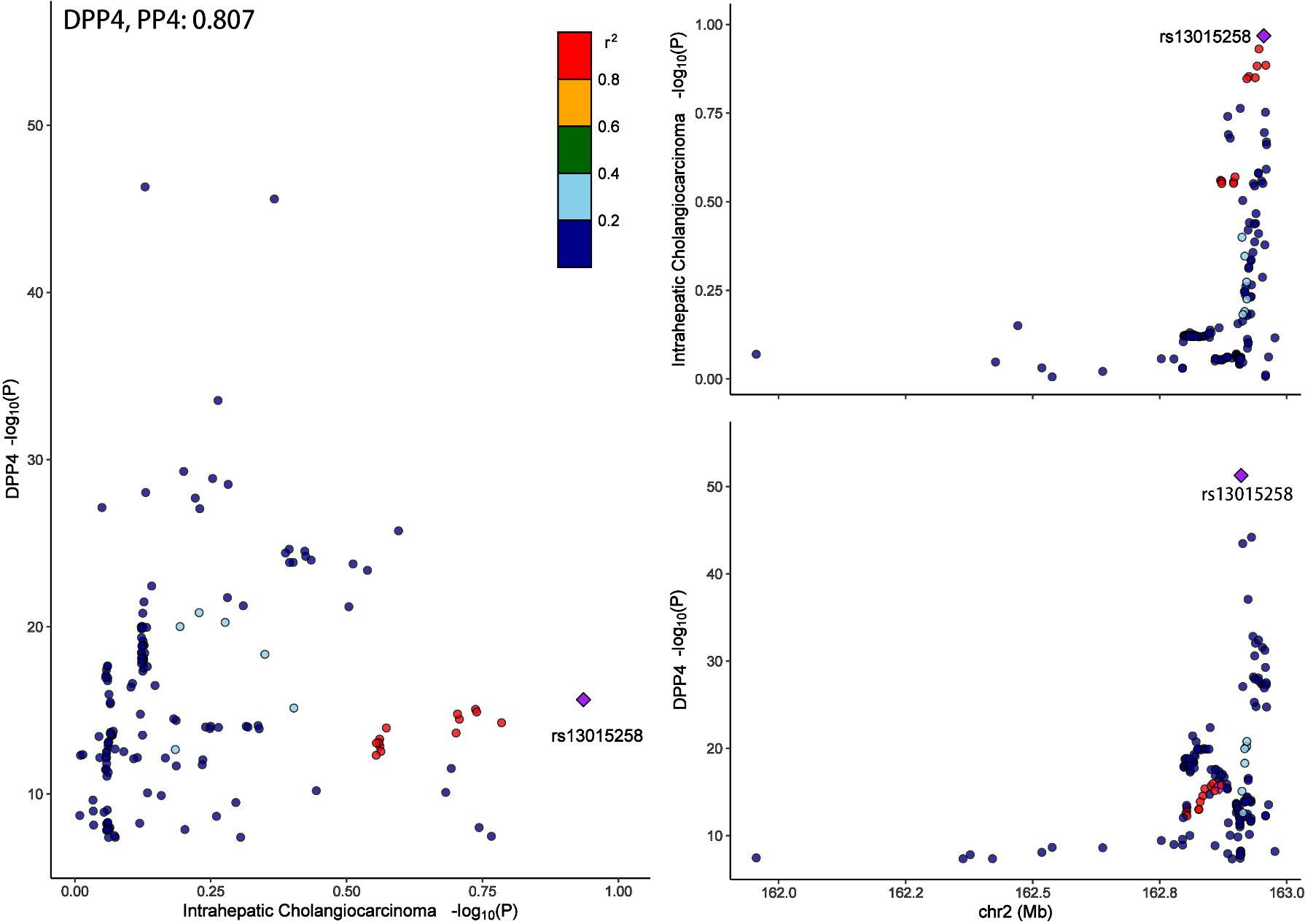

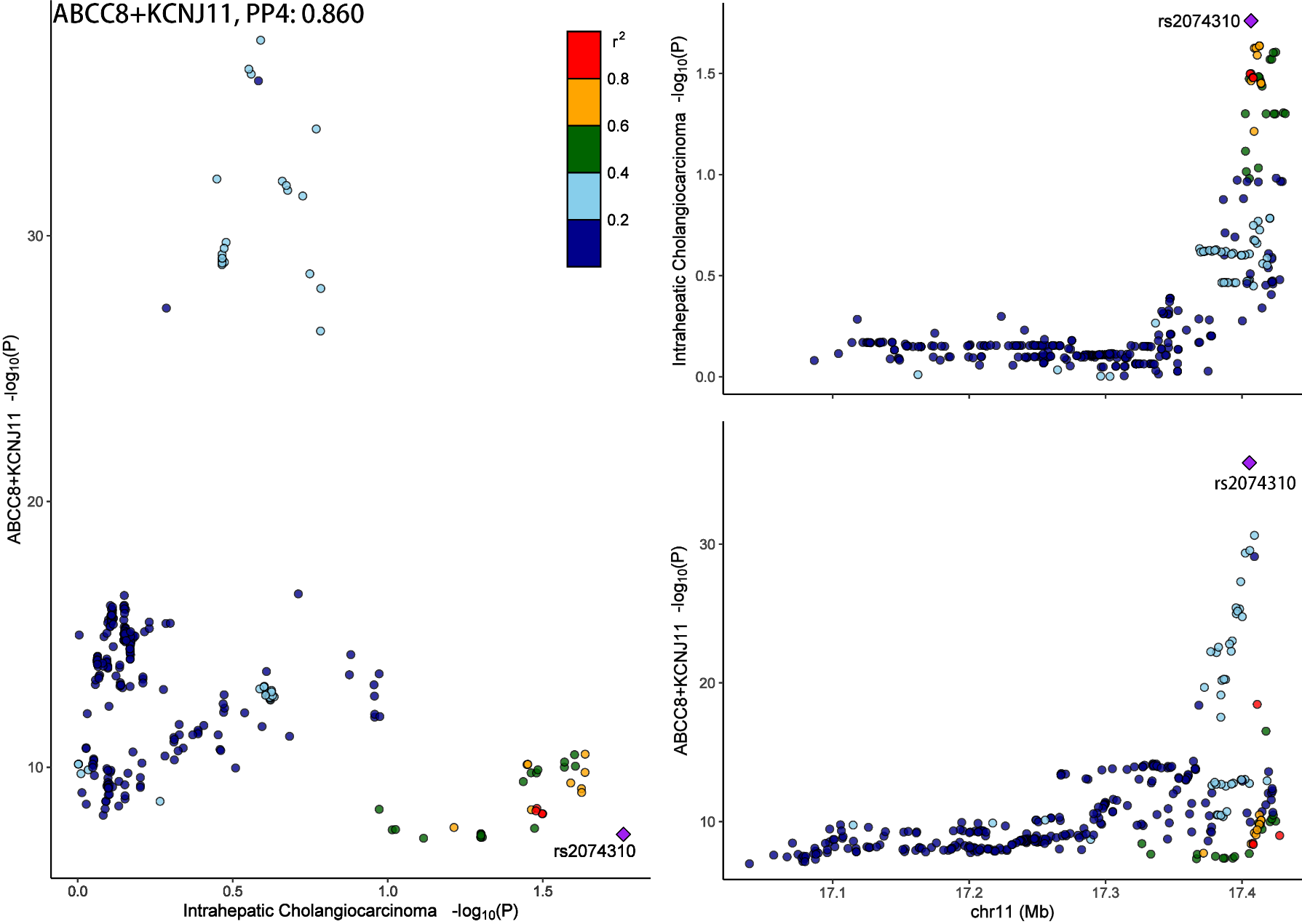

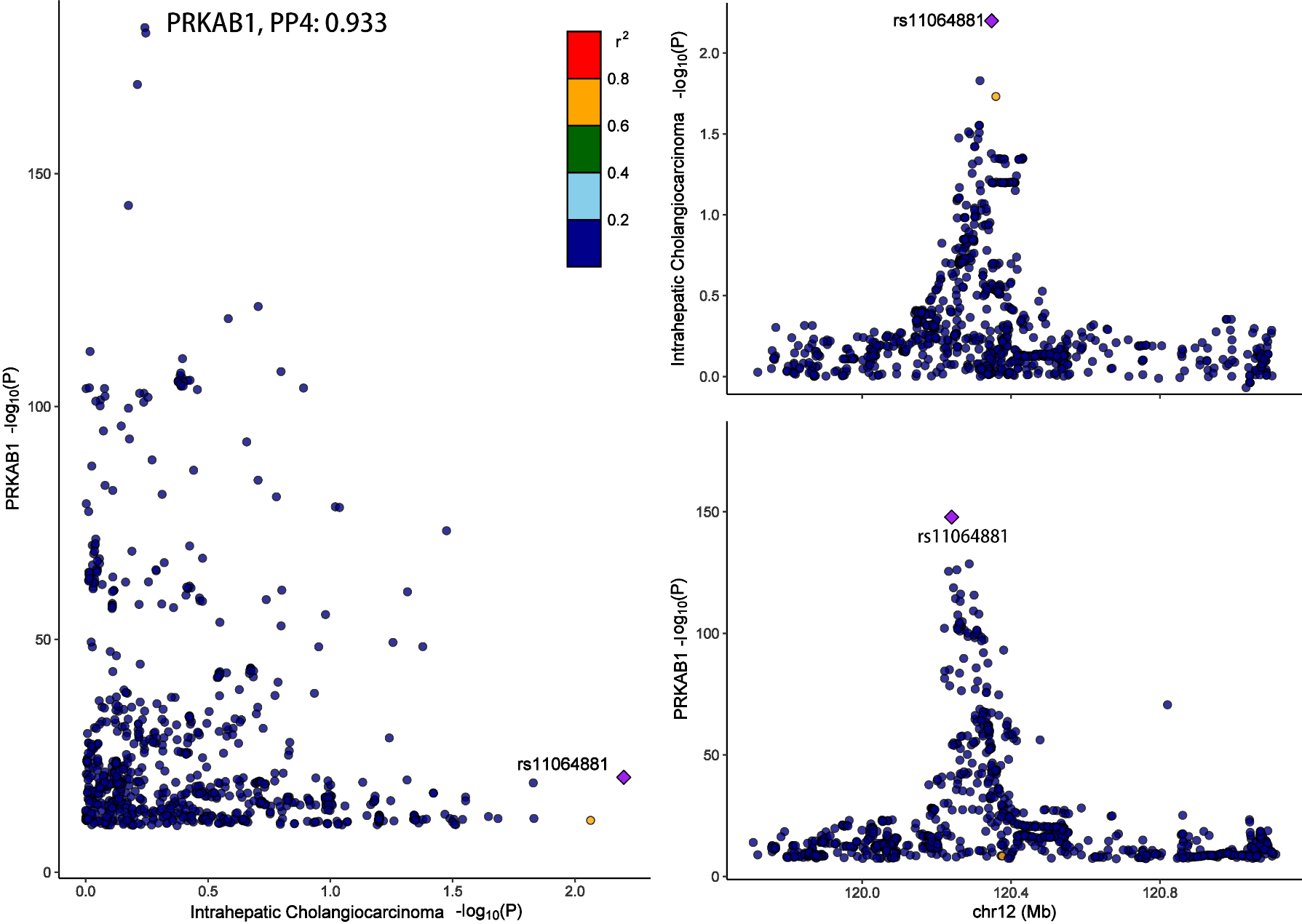

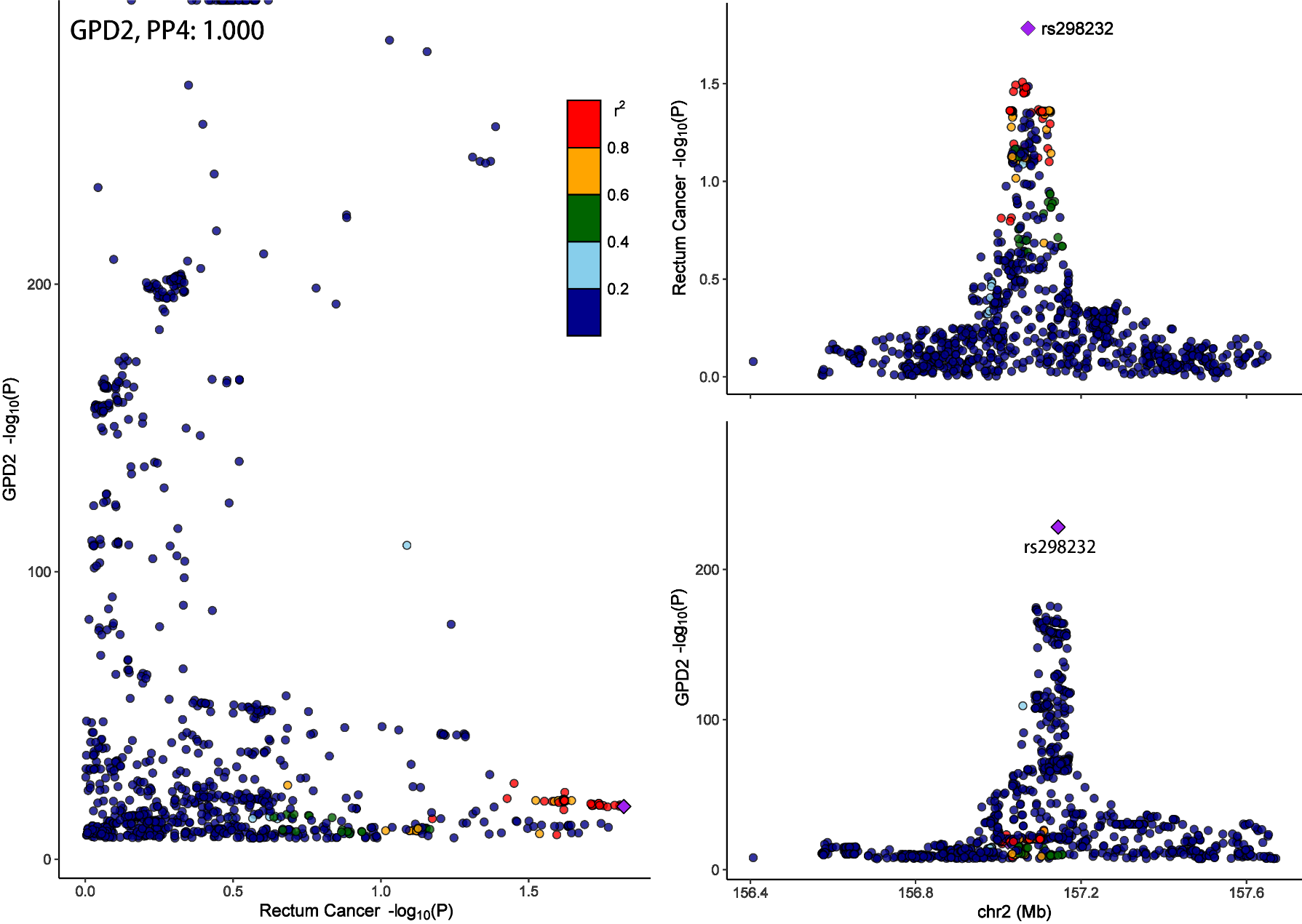

Supplement: Supplementary file 1 — Supplementary Material 1: The details of supplementary tables S1-S21 and supplementary figures S1-S8 [file 13578_2024_1214_MOESM1_ESM.docx]
